# The prevalence of molar-incisor hypomineralization: a systematic review and meta-analysis

**DOI:** 10.1038/s41598-021-01541-7

**Published:** 2021-11-17

**Authors:** Luísa Bandeira Lopes, Vanessa Machado, Paulo Mascarenhas, José João Mendes, João Botelho

**Affiliations:** 1 Clinical Research Unit (CRU), Centro de Investigação Interdisciplinar Egas Moniz, Egas Moniz – Cooperativa de Ensino Superior, CRL, Campus Universitário, Quinta da Granja, Monte de Caparica, 2829-511 Almada, Portugal; 2 Evidence-Based Hub, Centro de Investigação Interdisciplinar Egas Moniz, Egas Moniz – Cooperativa de Ensino Superior, CRL, 2829-511 Almada, Portugal

**Keywords:** Oral diseases, Diseases, Health care, Medical research, Oral manifestations

## Abstract

Molar-Incisor Hypomineralization (MIH) is a qualitative defect of enamel of unknown etiology, affecting one or more permanent molars and may include incisors. This condition is a clinical challenge and its prevalence is still uncertain given the recent increase in research. Thus, we aimed to comprehensively estimate the overall prevalence of MIH and associated characteristics. This systematic review is reported according to the Preferred Reporting Items for Systematic Reviews and Meta-Analysis (PRISMA). We searched articles using PubMed, MEDLINE, CENTRAL, Web of Science, SciELO, LILACS and TRIP databases, until July 2021. Heterogeneity and publication bias were computed via I^2^ test statistics and Egger’s significance test, respectively. Random-effects meta-analysis of prevalence were processed. We used the Strength of Recommendation Taxonomy [SORT] to grading the strength of evidence. Overall, 116 observational studies were included, with one study with moderate methodological quality and the remaining of high methodological quality. Subgroup analysis confirmed an influence of not using the 2003 MIH case definition (p = 0.0066). The pooled prevalence of MIH was 13.5% (95% CI 12.0–15.1, I^2^ = 98.0%). Affected incisors were seen in 36.6% (95% CI 30.0–43.7, I^2^ = 92.5%) of the cases. Lastly, the prevalence of hypomineralization of the second primary molars was observed in 3.6% of the MIH cases (95% CI 1.9–6.8, I^2^ = 96.3%). America was the continent with highest prevalence (15.3, 95% CI 12.8–18.3, p < 0.001, I^2^ = 96.3%) and Asia had the lowest prevalence (10.7, 95% CI 8.5–13.5, p < 0.001, I^2^ = 98.7%), however no continental differences were found. Sample size and year of publication were slight contributing factors to the heterogeneity in the analysis. Overall, these results were classified with a SORT A recommendation.

## Introduction

Molar-Incisor Hypomineralization (MIH) is designated as a qualitative defect of unknown etiology in the enamel development^1,2^. Since 2003, the European Academy of Pediatric Dentistry (EAPD) has proposed its first nomenclature to define a pathology of unknown etiology that affects one or more permanent molars and may include permanent incisors^1^.

As a potential oral public health concern, the prevalence of MIH became imperative to determine as a measure of interest in oral health programs. The prevalence of MIH was reported to range 2.8 to 40.2%, yet this inconsistency leads to a challenging interpretation and is mainly caused by the lack of standardization among clinicians/researchers^3^. As a result, the EAPD introduced a diagnostic and classification system for MIH, with the purpose of improving epidemiological assessments^3,4^.

Two systematic reviews have estimated the prevalence of MIH between 13.1% and 14.2, with significant variances amid regions^5,6^. Moreover, Schwendicke et al.^5^ estimated MIH prevalence on country scale via the Global Burden of Disease database, which may explain the variances between those regions. Additionally, both searches were conducted until mid 2017, and ever since, a number of new epidemiological studies have been published. However, other relevant information remains to be elucidated, namely the prevalence of moderate/severe cases, prevalence of molars and incisors affected and the prevalence of hypomineralization of the second primary molars (HSPM). For these reasons, conducting a new systematic review on the topic would be convenient and relevant globally.

In this sense, and given the increase research on the prevalence of MIH, we aimed to comprehensively investigate the global prevalence of MIH, as well as its associated characteristics.

## Methods

### Protocol and registration

The protocol for this systematic review was defined by all authors and registered at the National Institute for Health Research PROSPERO, International Prospective Register of Systematic Review (http://www.crd.york.ac.uk/PROSPERO, ID Number: CRD42021229435). We based our review design following the Preferred Reporting Items for Systematic Reviews and Meta-Analysis (PRISMA) guideline^7^.

### Focused question and eligibility criteria

We aimed to answer the following PECO question: “What is the global prevalence of MIH?”. The respective statements were as follows: Clinical/Epidemiologic studies in humans (P, Population); Diagnosis of MIH (E, Exposure); No MIH (C, Comparison); Prevalence of MIH (O, Outcome).

The primary aim was the prevalence of MIH. The secondary aims were the prevalence of moderate/severe MIH cases, prevalence of molars and incisors affected and the prevalence of HSPM.

Studies were eligible for inclusion based on the following criteria: (1) Observational studies reporting the prevalence of MIH; (2) Studies with clear reporting of MIH definition; (3) Subjects with no systemic disorders; (4) Studies including both genders.

In contrast, studies based on specific population, for example, children born preterm, studies which only reported on primary molars, and studies which focused on non-representative samples (e.g., institutionalized populations, particular professions, those with specific dental outcomes like high caries experience, among others) were excluded.

### Search strategy

Identification of studies for this systematic review was performed through detailed search strategies developed for each database (Pubmed, MEDLINE, CENTRAL [The Cochrane Central Register of Controlled Trials], Web of Science, SciELO [Scientific Electronic Library Online], EMBASE [The Excerpta Medica Database], LILACS [Latin-American scientific literature in health sciences], and TRIP [Turning Research Into Practise]) up to July 2021. Our search strategy was based on the following algorithm: "(hypomineralization OR hypomineralisation OR hypomineralized OR hypomineralized OR hypoplasia OR demarcated OR opacities OR MIH OR cheese molars) AND (survey OR questionnaire OR cross-sectional OR prevalence OR frequency OR population OR sample OR sampling) AND (molar OR molars OR incisors)".

### Study selection

Study selection was assessed independently by two investigators (LBL and JB), who performed the assessment of titles and/or abstracts of retrieved studies. For measurement reproducibility purposes, inter-examiner reliability following full-text assessment was calculated via kappa statistics. Any disagreements were resolved by discussion with a third author (VM).

### Data extraction process and data items

Data extraction was performed by two reviewers independently and in duplicate (LBL and JB). The agreement between the reviewers was assessed by Kappa statistics. Any paper classified as potentially eligible by either reviewer was ordered as a full text and independently screened by the reviewers. All disagreements were resolved through discussion with a third reviewer (VM). The following information was gathered in general description, research characteristics, methodology, and outcome measurements. The following standard information was extracted from each eligible study: first author’s name, year of publication, year of study conduct, country and place (region, city) of sampling, setting of sampling, sampling strategy, case definition, setting, observation setting, sample size, age of participants, total sample size, prevalence estimation, sex-specific sample size and prevalence (if available), the diagnostic criteria of MIH, mean number of affected teeth, and funding. Also, severity of MIH was registered whenever studies reported it. We considered studies that have defined or used classifications that considered severe cases of MIH as having demarcated enamel opacities with breakdowns, caries, persistent/spontaneous hypersensitivity affecting function and strong aesthetic concerns.

We applied no publication year nor language restrictions. Grey literature was searched via http://www.opengrey.eu/. If not reported, corresponding authors were contacted to obtain baseline data.

### Risk of bias (RoB) assessment

The Newcastle–Ottawa (NOS) Scale for case–control studies was used by two calibrated reviewers (LBL and JB). For calibration purposes, a random sample of 10 studies was assessed and reassessed 2 days later (to calculate Cohen's kappa). We have categorized studies as of low RoB (with 7–9 stars), moderate RoB (studies with 5–6 stars), and high RoB (with less than 5 stars) (as previously performed^8,9^). If any doubt occurred, they were resolved by discussion with a third author (VM).

### Summary measures and synthesis of results

We began by conducting a prior sensitivity analysis to understand if studies reporting MIH with the 2003 case definition would differ from other alternative case definitions. Predefined tables were prepared to collect continuous data, mean values and standard deviations (SD). Random-effects meta-analysis and forest plots of prevalence were calculated in R version 3.4.1 (R Studio Team 2018) using ‘meta’ package^10^, through DerSimonian-Laird random-effects meta-analysis. Subgroup meta-analysis was conducted for two reasons: (a) comparing the EAPD case definition with other alternative methods; (b) comparing continental prevalence of MIH. Also, a meta-analysis of binary outcome data comparing females and males prevalence was performed. Heterogeneity and publication bias were computed via I^2^ test statistics (p < 0.1) and Egger’s significance test, respectively^11^. Substantial heterogeneity was considered when I^2^ statistics exceeded 50%^11^. In meta-analysis with 10 or more studies included, we analyzed publication bias^11^. Meta-regressions were conducted using continuous variables to appraise potential sources of heterogeneity, such as sample size, female/male ratio (FMR), geographic location (latitude and longitude) and year of publication. The regression approach also allowed to quantify the percentage of heterogeneity that could be explained by that variable. All tests were two-tailed with alpha set at 0.05 except for the homogeneity test whose significance level cutoff was 0.10 due to the low power of the χ^2^ test with a limited number of studies. Estimates were described with 95% confidence interval (CI).

### Additional analyses

We employed the Strength of Recommendation Taxonomy (SORT) to appraise the strength and quality of the evidence^12^.

## Results

### Study selection

**Figure 1:**
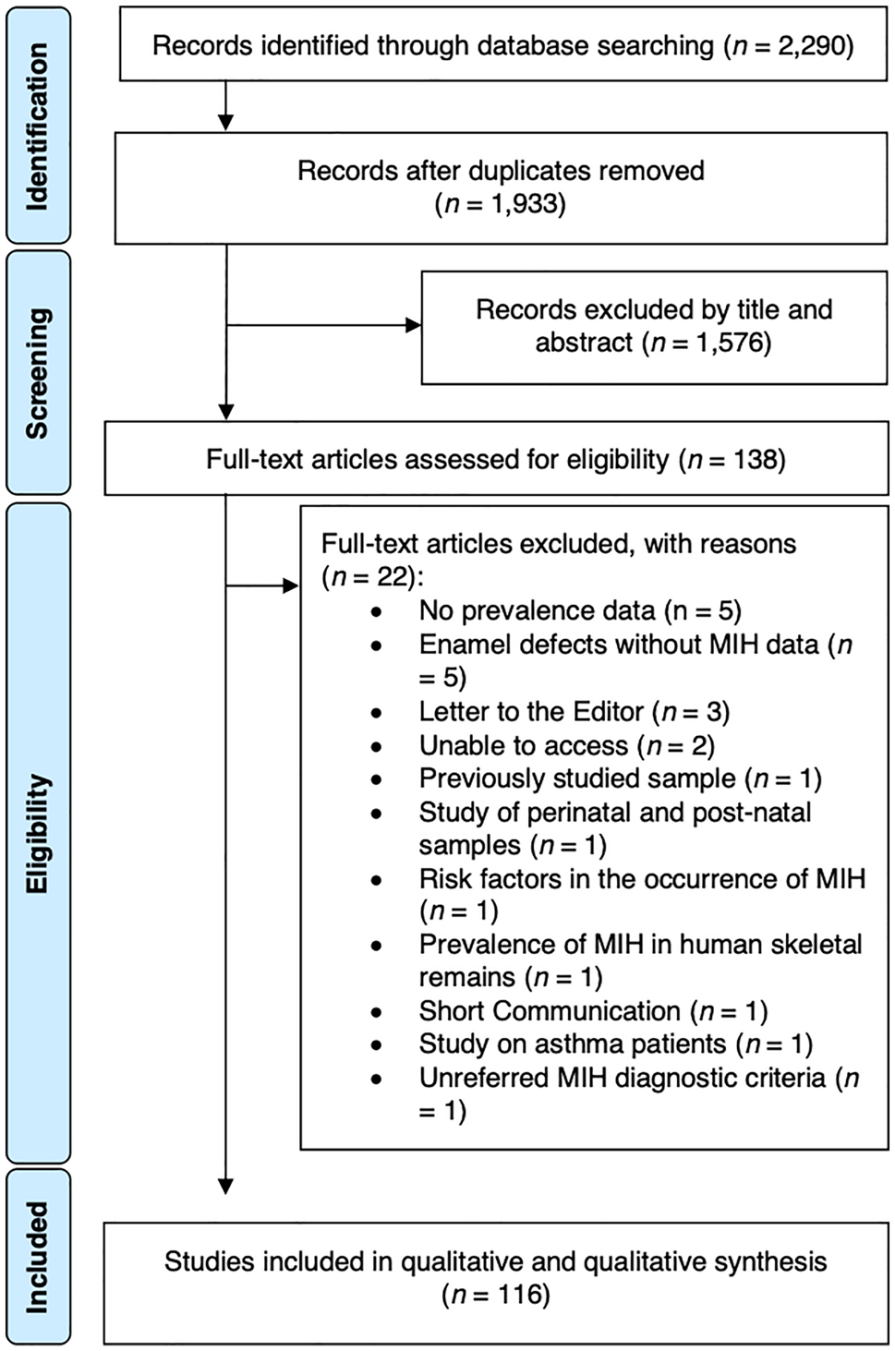
PRISMA flowchart.

The online search retrieved strategy 2290 possibly relevant publications. After duplicates removal, 357 papers were judged against the eligibility criteria, and 1576 were excluded after titles and/or abstracts review. Among 138 articles assessed for full paper review eligibility, 22 articles were excluded with the respective reasons for exclusion detailed in the Supplementary S2. As a result, a final number of 116 observational studies were included for qualitative synthesis (Fig. 1). Inter-examiner reliability at the full-text screening was considered very substantial (kappa score = 0.915, 95% CI 0.895–0.925).

### Studies characteristics

The characteristics of the included studies are presented in Table 1. We identified 116 different cohorts^13–128^ from fifty different countries, across five continents. The year of publication of the included studies ranged between 2003 and 2021. Overall, a total of 135,181 participants were included in this review, being 52,876 girls and 52,872 boys, even though 18 manuscripts did not report on sex distribution. Thirty-four papers did not report the prevalence of MIH according to sex. Seven studies reported data on HSPM. Most studies recorded the MIH-related hypomineralization according to the diagnostic criteria of the EAPD case definition^1^, and others indices were also used such as the modified Developmental Defects of Enamel (mDDE) index^129^, the Kemoli^88^, Mathu-Muju and Wright^130^ criteria, and the diagnostic criteria of Cho et al.^17,92^.

**Table 1:** Characteristics of the included studies.

Authors (year) (country)	Funding	Age range (years)	MIH classification	Total (MIH/No MIH) (n)	Females (n)		Males (n)		Molars affected (%)				Incisors affected (%)	Incisors and molars affected (%)	HSPM cases (n)
					MIH	Total	MIH	Total							
									1	2	3	4			
Abdalla et al. (2021) (Sudan)	None	8–11	EAPD^1^	568 (114/470)	55	284	59	284	33.3	29.8	23.7	13.2	7.6	12.5	NR
Ahmad et al. (2019) (United Arab Emirates)	NI	6–10	EAPD^1^	779 (59/720)	39	515	20	264	11.9	47.5	25.4	15.3	25.4	25.4	NR
Ahmadi et al. (2012) (Iran)	NI	7–9	EAPD^1^	433 (55/378)	25	218	30	215	NR	NR	NR	NR	NR	NR	NR
Alhowaish et al. (2021) (Saudi Arabia)	NR	8–10	EAPD^1^	893 (362/531)	194	461	168	432	NR	NR	NR	NR	NR	NR	NR
Allazzam et al. (2014) (Saudi Arabia)	NI	8–12	EAPD^1^	267 (23/244)	10	133	13	134	21.7	34.8	8.7	34.8	65.2	67.5	NR
Amend et al. (2020) (Germany)	Self-funded	6–12	EAPD^1^	2103 (283/1820)	NR	1005	NR	1098	30.4	24.7	19.4	25.4	48.7	65.2	64
Arheiam et al. (2021) (Saudi Arabia)	NI	8–10	EAPD^1^	1047 (162/885)	78	550	84	497	NR	NR	NR	NR	49.4	NR	NR
Arslanagic-Muratbegovic et al. (2020) (Bosnia and Herzegovina)	NI	6–9	EAPD^1^	444 (51/393)	28	NR	23	NR	11.8	35.3	23.5	35.3	64.7	64.7	NR
Balmer et al. (2011)/(2015) (England)	NI	12	mDDE^129^	3233 (514/2719)	NR	NR	NR	NR	NR	NR	NR	NR	NR	NR	NR
Bhaskar et al. (2014) (India)	NI	8–13	EAPD^1^	1173 (111/1062)	47	536	64	637	6.3	27.0	17.1	49.6	29.0	NR	NR
Biondi et al. (2011) (Argentina)	NI	NR	Mathu-Muju and Wright^130^	1098 (175/923)	NR	577	NR	521	NR	NR	NR	NR	18.9	NR	NR
Biondi et al. (2012) (Argentina and Uruguay)	NI	7–17	Mathu-Muju and Wright^130^	512 (32/480)	29	519	36	456	NR	NR	NR	NR	24.6	NR	NR
				463 (33/430)	29	519	36	456	NR	NR	NR	NR	26.1	NR	NR
Buchgraber et al. (2017) (Austria)	Medical University Graz	6–12	EAPD^1^	1111 (78/1033)	40	564	38	547	24.4	16.7	23.1	35.7	NR	NR	NR
Calderara et al. (2005) (Italy)	European Union, Regione Lombardia and Academy of Finland	7.3–8.3	EAPD^1^	227 (39/188)	NR	113	NR	114	NR	NR	NR	NR	NR	NR	NR
Cho et al. (2008) (Hong Kong)	NR	11–14	Cho et al. criteria^92^	2635 (73/2562)	NR	NR	NR	NR	49.3	24.7	15.1	11.0	33.0	45.0	NR
Da Costa-Silva et al. (2010) (Brazil)	NR	6–12	EAPD^1^	918 (182/736)	92	508	90	410	71	NR	NR	24	NR	55.2	NR
Dantas-Neta et al. (2016) (Brazil)	Piauí Research Foundation	11–14	EAPD^1^	594 (109/485)	NR	NR	NR	NR	NR	NR	NR	NR	NR	NR	NR
Dantas-Neta et al. (2018) (Brazil)	Piauí Research Foundation	8–10	EAPD^1^	744 (186/558)	103	412	83	332	NR	NR	NR	NR	NR	NR	NR
Davenport et al. (2019) (USA)	Marquette University	7–12	EAPD^1^	375 (36/339)	25	226	11	142	52,8	33,3	5.6	8.3	52.8	52.8	NR
De Lima et al. (2015) (Brazil)	State of Piauí Research Foundation	11–14	EAPD^1^	594 (109/485)	69	375	40	219	NR	NR	NR	NR	NR	NR	NR
Dietrich et al. (2003) (Germany)	NI	10–17	mDDE^129^	2408 (135/2273)	NR	NR	NR	NR	34.1	28.1	9.7	28.1	23.0	23.0	NR
Dourado et al. (2020) (Brazil)	NR	8–14	EAPD^1^	251 (117/134)	55	116	62	135	NR	NR	NR	NR	NR	NR	NR
Elfrink et al. (2012) (The Netherlands)	Erasmus MC, the Netherlands Organization for Health Research and Development and GABA	6–10	EAPD^1^	2530 (203/2327)	NR	NR	NR	NR	NR	NR	NR	NR	NR	NR	NR
Elzein et al. (2019) (Lebanon)	NR	7–9	EAPD^1^	659 (176/483)	96	NR	80	NR	12.8	19.5	26.8	40.9	45.1	45.1	NR
Emmaty et al. (2020) (India)	None	8–15	EAPD^1^	5318 (216/5102)	96	2613	120	2705	NR	NR	NR	NR	NR	NR	NR
Farias et al. (2020) (Brazil)	Paraíba State Research Support Foundation	8–10	EAPD^1^	471 (46/425)	26	265	20	206	NR	NR	NR	NR	NR	NR	NR
Fatturi et al. (2020) (Brazil)	São Paulo Research Foundation	8	EAPD^1^	731 (88/643)	39	357	49	374	NR	NR	NR	NR	NR	NR	NR
Fernandes et al. (2021) (Brazil)	NI	6–12	EAPD^1^	610 (60/550)	26	281	34	329	NR	NR	NR	NR	NR	NR	NR
Freitas Fernandes et al. (2021) (Brazil)	CAPES, National Council for Scientific and Technological Development (CNPq), Research Productivity Scholarship (302850/2016-3), and the State of Paraíba Research Support Foundation (FAPESQ/PB)	11–14	EAPD^1^	463 (50/413)	NR	293	NR	170	NR	NR	NR	NR	NR	NR	NR
Fteita et al. (2006) (Libya)	Academy of Finland	7–8,9	mDDE^129^	378 (11/367)	6	188	5	190	63.6	27.3	NR	9.1	NR	NR	NR
Gambetta-Tessini et al. (2018) (Australia)	NI	6–12	EAPD^1^	327 (48/279)	NR	NR	NR	NR	NR	NR	NR	NR	NR	NR	26
Gambetta-Tessini et al. (2019) (Chile)	Melbourne Dental School and Fund. Becas Chile	6–12	EAPD^1^	577 (91/486)	52	292	39	285	NR	NR	NR	NR	NR	NR	29
Garcia-Margarit et al. (2013) (Spain)	University of Valencia	8	EAPD^1^	840 (183/657)	NR	412	NR	428	NR	NR	NR	NR	32.5	NR	NR
Ghanim et al. (2011) (Iraq)	NI	7–9	EAPD^1^	823 (197/626)	NR	352	NR	471	NR	NR	NR	NR	NR	28.8	NR
Ghanim et al. (2013) (Iran)	Shiraz University of Medical Sciences	9–11	EAPD^1^	810 (164/646)	96	450	68	360	NR	NR	NR	NR	NR	NR	NR
Glodkowska et al. (2019) (Poland)	NI	6–12	EAPD^1^	1437 (51/1386)	27	726	24	711	3.9	17.0	21.0	58.0	NR	3.2	NR
Goswami et al. (2019) (India)	None	6–12	EAPD^1^	1026 (12/1014)	1	492	11	534	0	16.7	0	83.3	42.9	41.7	NR
Groselj et al. (2013) (Slovenia)	Slovenian Ministry of Science and Education	6.0–11.5	EAPD^1^	478 (102/376)	NR	212	NR	266	NR	NR	NR	NR	NR	NR	NR
Gurrusquieta et al. (2017) (Mexico)	NI	6–12	EAPD^1^	1156 (183/973)	NR	582	NR	574	NR	NR	NR	NR	NR	NR	NR
Hanan et al. (2015) (Brazil)	NI	6–10	EAPD^1^	2062 (188/1874)	90	941	98	933	NR	NR	NR	NR	NR	NR	NR
Hartsock et al. (2020) (USA)	University of Pittsburgh	7–32	EAPD^1^	104 (10/94)	8	64	2	40	NR	NR	NR	NR	NR	NR	NR
Heitmuller et al. (2013) (Germany)	Federal Ministry of Environment and the GABA GmBH	10	Koch et al.^140^	693 (253/2327)	NR	359	N R	334	NR	NR	NR	NR	NR	NR	NR
Hernández et al. (2018) (Spain)	NI	6–14	EAPD^1^	705 (56/649)	34	361	22	344	23.2	35.7	21.4	19.6	92.8	NR	NR
Hussain et al. (2018) (United Arab Emirates)	NI	6–12	EAPD^1^	342 (93/249)	70	215	23	127	NR	NR	NR	NR	NR	NR	NR
Hussein et al. (2015) (Malaysian)	Research Management Institute of Universiti Teknologi MARA	7–12	EAPD^1^	154 (26/128)	NR	87	NR	67	NR	NR	NR	NR	NR	50.0	NR
Hysi et al. (2016) (Albania)	NR	8–10	EAPD^1^	1575 (227/1348)	114	744	113	831	NR	NR	NR	NR	NR	NR	NR
Irigoyen-camacho et al. (2019) (Mexico)	NI	6–8	EAPD^1^	232 (47/185)	19	115	28	117	NR	NR	NR	NR	NR	NR	NR
				317 (101/216)	52	171	49	146	NR	NR	NR	NR	NR	NR	NR
Jancovik et al. (2014) (Bosnia and Herzegovina)	NI	8	EAPD^1^	141 (26/115)	NR	70	NR	71	NR	NR	NR	NR	NR	NR	NR
Jasulaityte et al. (2007) (Lithuania)	NI	6–9	EAPD^1^	1227 (190/1087)	102	629	88	560	NR	NR	NR	NR	NR	44.4	NR
Jasulaityte et al. (2008) (Netherlands)	NI	9	EAPD^1^	442 (63/379)	NR	220	NR	222	11.1	30.2	22.2	36.5	2.6	NR	NR
Jeremias et al. (2013) (Brazil)	Federal funding from São Paulo State	6–12	EAPD^1^	1157 (142/1015)	88	622	54	535	23.9	NR	NR	NR	51.4	51.4	NR
Jurlina et al. (2020) (Croatia)	None	8	EAPD^1^	729 (88/641)	49	356	39	373	NR	NR	NR	NR	NR	6.6	NR
Kemoli et al. (2009) (Kenya)	NI	NR	Kemoli^88^	3591 (493/3098)	375	1593	118	1998	NR	NR	NR	NR	NR	NR	NR
Kevrekidou et al. (2015) (Greece)	NI	8–14	EAPD^1^	2335 (498/1837)	253	1196	245	1139	48.0	28.0	13.0	11.0	NR	54.0	NR
Kilinç et al. (2019) (Turkey)	NI	9–10	EAPD^1^	1237 (142/1095)	69	NR	73	NR	NR	NR	NR	23.4	NR	NR	NR
Kirthiga et al. (2015) (India)	NI	11–16	Cho et al.^92^	2000 (179/1821)	92	827	87	1173	NR	NR	NR	NR	NR	NR	NR
Kohlboeck et al. (2013) (Germany)	Federal Ministry of Environment and the GABA GmBH	10	EAPD^1^	1126 (381/745)	NR	549	NR	577	NR	NR	NR	NR	NR	NR	NR
Koruyucu et al. (2018) (Turkey)	Istanbul University	8 and 11	EAPD^1^	1511 (215/1296)	113	751	102	760	NR	NR	NR	NR	NR	NR	NR
Krishnan et al. (2015) (India)	NI	8–13	EAPD^1^	4989 (384/4605)	253	2831	131	2158	NR	NR	NR	NR	NR	NR	NR
Kühnisch et al. (2018) (Germany)	Federal Ministry for Education	15	EAPD^1^	1302 (224/1078)	NR	651	NR	651	38.2	NR	NR	17.1	NR	9.8	NR
Kuscu et al. (2009) (Turkey)	NI	NR	EAPD^1^	153 (14/139)	6	72	8	67	NR	21.4	NR	NR	NR	50.0	NR
López Jordi et al. (2014) (Argentina & Uruguay)	NI	7–17	Mathu-Muju and Wright^130^	1090 (176/914)	NR	572	NR	518	NR	NR	NR	NR	NR	NR	NR
				626 (77/549)	NR	328	NR	298	NR	NR	NR	NR	NR	NR	NR
Lygidakis et al. (2008) (Greece)	NI	5.5–12	EAPD^1^	3518 (360/3158)	211	NR	149	NR	NR	NR	NR	NR	NR	62.5	NR
Mahoney et al. (2009) (New Zealand)	NR	7–10	mDDE^129^	234 (44/190)	NR	117	25	117	NR	NR	NR	NR	NR	NR	NR
Mahoney et al. (2011) (New Zealand)	NR	7–10	mDDE^129^	522 (78/444)	NR	282	NR	240	NR	NR	NR	NR	NR	NR	NR
Martínez Gomez et al. (2012) (Spain)	NI	6–14	EAPD^1^	505 (90/415)	45	246	45	259	10.0	11.1	11.1	8.8	NR	58.8	NR
Martinovic et al. (2017) (Kosovo)	NR	8 and 10	EAPD^1^	712 (87/625)	49	383	38	329	NR	NR	NR	NR	NR	100.0	NR
Mejia et al. (2019) (Colombia)	NI	6–15	EAPD^1^	1075 (120/955)	46	443	74	632	NR	NR	NR	NR	NR	NR	NR
Mishra et al. (2016) (India)	None	8–12	EAPD^1^	1369 (191/1178)	99	NR	92	NR	NR	NR	NR	NR	NR	27.7	NR
Mittal et al. (2013) (India)	NI	6–9	EAPD^1^	1792 (113/1679)	50	NR	63	NR	NR	NR	NR	NR	NR	NR	NR
Mulic et al. (2017) (Bosnia and Herzegovina)	University of Oslo	8–9	EAPD^1^	103 (12/91)	NR	41	NR	62	25	50	25	NR	NR	NR	NR
Muratbegovic et al. (2007) (Bosnia and Herzegovina)	NI	12	EAPD^1^	560 (69/491)	36	NR	33	NR	NR	NR	NR	NR	NR	92.5	NR
Negre-Barber et al. (2016) (Spain)	Spanish national R&D&I Plan and European Regional Development Fund	8–9	EAPD^1^	414 (100/314)	46	202	54	212	17.0	22.0	26.0	35.0	60.0	NR	60
Ng et al. (2014) (Singapore)	NI	NR	EAPD^1^	1083 (135/948)	68	608	67	475	46.7	22.2	8.1	4.4	25.2	3.2	23
Ordonez-Romero et al. (2021) (Ecuador)	None	7–12	EAPD^1^	249 (23/226)	17	144	6	105	NR	NR	NR	NR	25.6	NR	NR
Oyedele et al. (2015) (Nigeria)	NI	8–10	EAPD^1^	469 (83/386)	32	214	51	255	NR	NR	NR	NR	NR	NR	NR
Padavala et al. (2018) (India)	None	7–12	EAPD^1^	170 (22/148)	7	85	15	85	NR	NR	NR	NR	10.8	40.9	NR
Parikh et al. (2012) (India)	NI	8–12	EAPD^1^	1366 (126/1240)	58	NR	68	NR	NR	NR	NR	NR	NR	82.5	NR
Petrou et al. (2014)/(2015) (Germany)	NI	7–10	EAPD^1^	2395 (242/2153)	114	1200	128	1195	39.2	NR	NR	NR	42.2	NR	NR
Pitiphat et al. (2014) (Thailand)	Thailand Research Fund	6–7	EAPD^1^	484 (95/389)	51	246	44	238	86.0	NR	NR	NR	NR	NR	NR
Portella et al. (2019) (Brazil)	CAPES Grant/Award Number: 001	8	EAPD^1^	728 (88/640)	NR	356	NR	372	NR	NR	NR	NR	54.5	NR	NR
Preusser et al. (2007) (Germany)	NR	6–12	Koch et al.^140^	1002 (59/943)	NR	496	NR	506	NR	NR	NR	NR	NR	NR	NR
Rai et al. (2018) (India)	NI	7–9	mDDE^129^	992 (212/780)	80	460	132	532	NR	NR	NR	NR	NR	NR	NR
Rai et al. (2019) (India)	Indian Council of Medical Research	9–12	EAPD^1^	1600 (210/1390)	104	814	106	786	NR	NR	NR	NR	12.1	70.2	NR
Ray et al. (2020) (India)	None	8–12	EAPD^1^	1525 (87/1438)	37	725	50	800	NR	NR	NR	NR	56.3	18.4	NR
Reyes et al. (2019) (Brazil)	NI	8	EAPD^1^	731 (88/643)	39	357	49	374	NR	NR	NR	N R	6.6	NR	NR
Rodrigues et al. (2015) (Brazil)	NI	7–14	mDDE^129^	1179 (30/1149)	NR	NR	NR	NR	NR	NR	NR	NR	NR	NR	NR
Saber et al. (2018) (Egypt)	NI	8–12	EAPD^1^	1001 (23/978)	14	502	9	499	NR	NR	NR	NR	NR	NR	NR
Saitoh et al. (2018) (Japan)	Japanese Dental Science Federation	7–9	EAPD^1^	4496 (892/3604)	464	2280	428	2216	NR	NR	NR	NR	NR	NR	NR
Sakly et al. (2020) (Tunisia)	None	7–12	EAPD^1^	510 (181/329)	82	257	99	253	NR	NR	NR	NR	NR	NR	NR
Schmalfuss et al. (2015) (Norway)	NI	16	EAPD^1^	794 (110/684)	NR	380	NR	414	48.2	30.0	12.7	9.1	41.8	NR	NR
Shrestha et al. (2015) (Nepal)	NI	7–12	EAPD^1^	747 (102/645)	48	357	54	288	4.9	9.8	10.8	74.5	84.3	85.3	NR
Sidhu et al. (2019) (Canada)	Hospital for Sick Children	NR	EAPD^1^	429 (29/400)	NR	181	NR	248	NR	NR	NR	NR	NR	NR	19
Silva et al. (2020) (Brazil)	Coordenação de Aperfeiçonamento de Pessoal de Nivel Superior Brasil—(CAPES)	7–14	EAPD^1^	407 (59/348)	26	182	33	225	NR	NR	NR	NR	NR	NR	NR
Silva Júnior et al. (2015) (Brazil)	Federal University of Pará	5–17	EAPD^1^	260 (23/237)	11	112	12	148	NR	NR	NR	NR	39.1	34.8	NR
Singh et al. (2020) (India)	None	7–10	EAPD^1^	649 (97/552)	NR	NR	NR	NR	5.7	39.3	7.4	47.5	93.8	8.8	NR
Sonmez et al. (2013) (Turkey)	NI	7–12	EAPD^1^	4018 (308/3710)	156	2029	152	2020	NR	NR	NR	NR	NR	NR	NR
Sosa-Soto et al. (2021) (Mexico)	Programa de Fortalecimiento de la Calidad Educativa	8	EAPD^1^	613 (76/537)	NR	295	NR	318	38.2	NR	NR	17.1	NR	NR	NR
Souza et al. (2013) (Brazil)	Federal Funding from São Paulo State	7–12	EAPD^1^	1151 (142/1009)	88	624	54	527	NR	NR	NR	NR	NR	NR	NR
Soviero et al. (2009) (Brazil)	State University of Rio de Janeiro	7–13	EAPD^1^	249 (100/149)	NR	NR	NR	NR	NR	NR	NR	NR	NR	NR	NR
Subramaniam et al. (2016) (India)	None	7–9	EAPD^1^	2500 (12/2488)	7	1104	5	1396	42.3	40.4	5.8	11.5	23.1	23.1	NR
Tagelsir Ahmed et al. (2020) (USA)	NI	6–15	EAPD^1^	337 (43/294)	24	169	19	168	NR	NR	NR	NR	NR	NR	6
Temilola et al. (2015) (Nigeria)	NI	NR	Kemoli^88^	236 (23/213)	14	120	9	116	NR	NR	NR	NR	NR	NR	8
Thakur et al. (2020) (India)	NR	8–16	EAPD^1^	2000 (58/1942)	NR	967	NR	1033	8.5	32.3	13.6	44.2	41.2	41.2	13
Tourino et al. (2016) (Brazil)	None	8–9	EAPD^1^	1181 (241/940)	125	599	116	582	NR	NR	NR	NR	NR	NR	NR
Villanueva-Gutierrez et al. (2019) (Mexico)	Metropolitan Autonomous University-Xochimilco	7–12	EAPD^1^	686 (243/443)	120	365	123	321	6.6	21.7	28.3	43.4	NR	NR	NR
Wogelius et al. (2008) (Danmark)	“Augustinus Foundation’’, the Danish Cancer Society, and Boernecancerfonden	6–8	EAPD^1^	647 (241/426)	116	321	125	326	32.0	27.4	13.7	27.0	NR	NR	NR
Wuollet et al. (2014) (Finland)	Academy of Finland	7–13	EAPD^1^	818 (140/678)	66	401	74	417	NR	NR	NR	NR	NR	NR	NR
Wuollet et al. (2016) (Finland)	Academy of Finland	NR	EAPD^1^	287 (33/254)	17	128	16	159	NR	NR	NR	NR	NR	NR	NR
Wuollet et al. (2018) (Finland)	Academy of Finland	8–13	EAPD^1^	636 (115/521)	NR	NR	NR	NR	NR	NR	NR	NR	NR	NR	NR
Yannam et. (2016) (India)	NI	8–12	EAPD^1^	2864 (277/2587)	NR	1365	NR	1499	NR	NR	NR	NR	NR	NR	NR
Yi et al. (2020) (China)	Scientific Research Fund of National Health Commission of China	12–15	EAPD^1^	6523 (655/5868)	340	3295	315	3228	NR	NR	NR	NR	28.4	28.4	NR
Zawaideh et al. (2011) (Jordania)	NI	7–9	EAPD^1^	3241 (570/2671)	302	1539	268	1702	41.0	28.0	20.0	11.0	32.0	32.0	NR

*NR* Not reported, *NI* No information, *EAPD* European Academy of Pediatric Dentistry (Weerheijm et al.^1^), *mDDE* modified Developmental Defects of Enamel index.

Three cohorts had their data reported in more than one article (Petrou et al.^78^ and Petrou et al.^109^; Balmer et al.^13^ and Balmer et al.^14^; Negre-Barber et al.^110^ and Negre-Barber et al.^111^); thus, these papers were grouped under a single name study as follows: Petrou et al.^78,109^; Balmer et al.^13,14^; and, Negre-Barber et al.^110,111^. Also, three studies reported in the same study two cohorts: Biondi et al.^16^ reported data for Buenos Aires (Argentina) and Montevideo (Uruguay); López Jordi et al.^108^ reported data for Buenos Aires (Argentina) and Montevideo (Uruguay); and Irigoyen-Camacho et al.^62^ reported data for both 2008 and 2017 cohorts.

### Assessment of RoB within studies

Inter-examiner reliability at RoB analysis was considered very substantial (kappa score = 0.885, 95% CI 0.865–0.905). The RoB for observational studies, with the NOS, ranged from 6 to 9 stars (Supplementary S3). After the assessment, forty-eight had the maximum score (9/9). Additionally, fifty-three and six articles scored 8/9 and 7/9, respectively. Only one paper was of moderate RoB (score = 6/9). The main sources of inconsistencies arose from the representativeness of the cases. While all articles succeed to apply an adequate MIH case definition, selection of control, ascertainment of exposure, equal method of assessment of cases and controls and non-response rate (100.0%, n = 113), studies failed to provide adequate representativeness of the cases (48.7%, n = 55), two studies failed the definition of controls (1.8%) and 8.8% only provided information regarding MIH and not any other variables (n = 10).

### Prevalence of MIH

A first subgroup meta-analysis confirmed that estimates from studies using the EAPD 2003 classification were significantly different from studies with alternative classifications (categorized as ‘others’) (p = 0.0061) (Supplementary S4). This initial analysis comprised 133,734 participants. Thus, we proceeded with the analyses using only studies reporting prevalence through the 2003 MIH case definition.

### Global prevalence (primary outcome)

The overall prevalence of MIH for a total of 113,089 participants was estimated at 13.5% (95% CI 12.1–15.1, p < 0.001) (Table 2), with high heterogeneity (I^2^ = 98.0%) (Supplementary S5). Cumulative meta-analysis confirmed the overall estimate was not influenced by a particular study or group of studies (Supplementary S6A). We further confirmed the non-existence of influential studies through leave-one-out meta-analysis (Supplementary S6B).

**Table 2:** Meta-analysis on the prevalence of MIH cases, severity of cases, number of affected molars, cases with affected incisors and HSPM.

Condition	N	Estimate (%)	95% CI (%)	p-value	I^2^ (%)	Egger test t (p-value)
**MIH**	98	13.5	12.0–15.1	< 0.001	98.0	− 2.366 (0.179)
**Moderate-to-severe cases**	33	36.3	29.9–43.2	< 0.001	95.2	0.233 (0.052)
**Number of affected molars**						
1	31	24.3	18.9–30.7	< 0.001	94.2	− 3.392 (0.002)
2	27	26.7	23.9–29.7	< 0.001	65.0	− 0.141 (0.889)
3	26	18.1	13.8–23.3	< 0.001	90.9	− 1.207 (0.239)
4	27	27.4	21.1–34.7	< 0.001	94.0	− 0.020 (0.984)
**Cases with affected incisors**	31	38.7	32.1–45.8	< 0.001	93.2	− 0.747 (0.461)
**Cases with both molars and incisors affected**	36	42.1	34.9–50.0	< 0.001	95.5	− 0.153 (0.774)
**HSPM**	7	3.6	1.9–6.8	< 0.001	95.9	–

*MIH* Molar-Incisor Hypomineralization, *HSPM* Hypomineralization of the Second Primary Molars, *95% CI* 95% Confidence Interval.

The prevalence of moderate to severe cases of MIH was estimated at 36.3% (95% CI 29.9–43.2, I^2^ = 95.2%) (Table 2, Supplementary S7). Detailed information on the definition of severity in each study was collectively presented in Table 3. Regarding the number of affected molars, estimates point to 24.3% of cases with one molar (95% CI 18.9–30.7, I^2^ = 94.2%), 26.7% of cases with two molars (95% CI 23.9–29.7, I^2^ = 65.0%), 18.1% of cases with three molars (95% CI 13.8–23.3, I^2^ = 90.0%) and 26.8% of cases with four molars (95% CI 21.1–34.7, I^2^ = 94.0%) (Supplementary S8-S11). The cases with affected incisors were estimated at 38.7% (95% CI 32.1–45.8, I^2^ = 93.2%) (Supplementary S12), while cases with both molars and incisors were estimated at 42.1% (95% CI 34.9–50.0, I^2^ = 95.5%) (Supplementary S13). Lastly, the prevalence of HSPM cases was estimated at 3.6% (95% CI 1.9–6.8, I^2^ = 96.3%) (Supplementary S14). All the latter results had high heterogeneity.

**Table 3:** Detailed case definition of MIH severity for each study with the respective reported prevalence.

Authors (year) (country)	Severity Index/definition	Definition	Moderate/severe cases (%)
Amend et al. (2020) (Germany)	Wetzel and Reckel scale^34^	*Degree 1* (isolated hypomineralization of white cream to yellow–brown color, solely located in the uppermost part of the tooth crown (chewing surface), no post-eruptive enamel breakdown); *degree 2* (enamel hypomineralization of yellow–brown color affecting almost all humps in the coronal part of the tooth crown combined with a small amount of post-eruptive enamel breakdown), and *degree 3* (extensive enamel hypomineralization of yellow–brown color along with extensive post-eruptive enamel breakdown causing changes of the tooth crown morphology)	78.4
Arslanagic-Muratbegovic et al. (2020) (Bosnia & Herzegovina)	–	≥ 1 tooth with post-eruptive enamel breakdown, atypical fillings or tooth extracted due to MIH	82.0
Da Costa-Silva et al. (2010) (Brazil)	Leppäniemi et al.^135^	*Mild* (demarcated opacities without fracture), *moderate* (hard and fractured enamel and need for treatment), and *severe* (loss of tooth structure affecting the enamel and dentine, replacement of hard tissues with atypical restorations, and tooth extraction due to hypomineralization)	54.0
Dantas-Neta et al. (2016) (Brazil)	Leppäniemi et al.^135^	*Mild* (demarcated opacities without fracture), *moderate* (hard and fractured enamel and need for treatment), and *severe* (loss of tooth structure affecting the enamel and dentine, replacement of hard tissues with atypical restorations, and tooth extraction due to hypomineralization)	50.5
Dantas-Neta et al. (2018) (Brazil)	Leppäniemi et al.^135^	*Mild* (demarcated opacities without fracture), *moderate* (hard and fractured enamel and need for treatment), and *severe* (loss of tooth structure affecting the enamel and dentine, replacement of hard tissues with atypical restorations, and tooth extraction due to hypomineralization)	5.4
Davenport et al. (2019) (USA)	–	*Mild* (demarcated opacities without enamel breakdown, occasional sensitivity to external stimuli) and *severe* (demarcated enamel with breakdown, caries, and persistent/ spontaneous hypersensitivity)	30.6
Ghanim et al. (2013) (Iran)	–	Mild (color changes only [i.e. creamy white or yellow/brown]), *moderate* (loss of enamel substance), and *severe* (loss of enamel associated with affected dentine and/or atypical restoration)	34.3
Glodkowska et al. (2019) (Poland)	Lygidakis et al.^134^	*Mild* (demarcated enamel opacities without enamel breakdown, occasional sensitivity to external stimuli but not brushing and only mild aesthetic concerns on discoloration of the incisors), and *severe* (demarcated enamel opacities with breakdowns, caries, persistent/spontaneous hypersensitivity affecting function and finally strong aesthetic concerns that may have socio-psychological impact)	26.6
Gurrusquieta et al. (2017) (Mexico)	Mathu-Muju and Wright^130^	*Mild* (Opacities delimited in areas free of occlusal forces, isolated opacities, no enamel loss in opaque areas, no history of dental hypersensitivity, no activities related to caries of affected enamel, alterations of incisors), *moderate* (atypical and intact restorations may be present, opacities delimited in the occlusal/incisal third of the tooth, without loss of the structure after eruption, loss of post-eruptive enamel and carious lesions that are limited to 1 or 2 areas, without participation of cusps, tooth sensitivity and often, aesthetic complaints) and *severe* (post-eruptive losses, history of tooth sensitivity, extensive carious lesions associated with the affected enamel, coronary destruction with pulp involvement, presence of defects in atypical restorations, aesthetic complaints)	43.7
Hartsock et al. (2020) (USA)	Lygidakis et al.^134^	*Mild* (demarcated enamel opacities without enamel breakdown, occasional sensitivity to external stimuli but not brushing and only mild aesthetic concerns on discoloration of the incisors), and **severe** (demarcated enamel opacities with breakdowns, caries, persistent/spontaneous hypersensitivity affecting function and finally strong aesthetic concerns that may have socio-psychological impact)	30.0
Hussain et al. (2018) (United Arab Emirates)	Chawla et al.^138^	Hypomineralisation Severity Index	47.0
Irigoyen-camacho et al. (2019) (Mexico)	–	*Mild *(demarcated opacities affected less than one-third of the tooth surface, without post-eruptive enamel breakdown), *moderate* (demarcated opacities that affected at least one-third but less than two-thirds of the surface, without post-eruptive enamel breakdown; atypical caries lesions could affect less than two-thirds of the surface), and *severe* (demarcated opacities that affected more than two-thirds of the tooth surface, or the presence of post-eruptive enamel breakdown, atypical caries lesions larger than two-thirds of the surface, or large restorations with unusual shape, extended to smooth surfaces, or extraction of the tooth because of MIH)	21.2
			30.7
Janković et al. (2014) (Bosnia and Herzegovina)	–	*Mild* (tooth enamel color changes [white, yellow or brown]), *moderate* (discoloration and minimal loss of tooth substances without the need for restoration), and *severe* (damaged enamel and dentin loss that require restoration)	13.4
Jasulaityte et al. (2008) (The Netherlands)	–	*Mild* (opacities) and *severe* (enamel breakdown and atypical restorations both include lesions with disintegrated enamel, in one case restored)	45.2
Jeremias et al. (2013) (Brazil)	Jasulaityte et al.^63^	*Severe* (post-eruptive enamel breakdown, atypical restorations and extraction due to MIH)	9.3
Kevrekidou et al. (2015) (Greece)	Lygidakis et al.^134^	*Mild* (demarcated enamel opacities without enamel breakdown, occasional sensitivity to external stimuli but not brushing and only mild aesthetic concerns on discoloration of the incisors), and *severe* (demarcated enamel opacities with breakdowns, caries, persistent/spontaneous hypersensitivity affecting function and finally strong aesthetic concerns that may have socio-psychological impact)	25.0
Kühnisch et al. (2018) (Germany)	Kühnisch et al.^69^	*Severe* (hypomineralization on first permanent molars and incisors)	56.7
Martínez Gomez et al. (2012) (Spain)	Mathu-Muju and Wright^130^	*Mild *(Opacities delimited in areas free of occlusal forces, isolated opacities, no enamel loss in opaque areas, no history of dental hypersensitivity, no activities related to caries of affected enamel, alterations of incisors), *moderate* (atypical and intact restorations may be present, opacities delimited in the occlusal/incisal third of the tooth, without loss of the structure after eruption, loss of post-eruptive enamel and carious lesions that are limited to 1 or 2 areas, without participation of cusps, tooth sensitivity and often, aesthetic complaints) and *sever***e** (post-eruptive losses, history of tooth sensitivity, extensive carious lesions associated with the affected enamel, coronary destruction with pulp involvement, presence of defects in atypical restorations, aesthetic complaints)	50.0
Martinovic et al. (2017) (Kosovo)	–	*Mild* (stained changes in the tooth enamel), *moderate* (changes in color [white/opaque, yellow or brown] and minimal loss of tooth substance with no need for restoration, or minimally invasive treatment is sufficient to repair defects), and *severe* (loss of damaged enamel and dentin which require restoration)	40.3
Mejia et al. (2019) (Colombia)	Leppäniemi et al.^135^	*Mild* (demarcated opacities without fracture), and *severe* (opacity with loss of structure compromising enamel and/or dentin, with atypical restorations, and/or exodontia due to hypomineralization)	15.0
Parikh et al. (2012) (India)	Lygidakis et al.^134^	*Mild* (demarcated enamel opacities without enamel breakdown, occasional sensitivity to external stimuli but not brushing and only mild aesthetic concerns on discoloration of the incisors), and *sever***e** (demarcated enamel opacities with breakdowns, caries, persistent/spontaneous hypersensitivity affecting function and finally strong aesthetic concerns that may have socio-psychological impact)	22.3
Petrou et al. (2014)/(2015) (Germany)	Lygidakis et al.^134^	*Mild* (demarcated enamel opacities without enamel breakdown, occasional sensitivity to external stimuli but not brushing and only mild aesthetic concerns on discoloration of the incisors), and **severe** (demarcated enamel opacities with breakdowns, caries, persistent/spontaneous hypersensitivity affecting function and finally strong aesthetic concerns that may have socio-psychological impact)	52.1
Portella et al. (2019) (Brazil)	Leppäniemi et al.^135^	*Mild *(demarcated opacities without fracture), *moderate* (hard and fractured enamel and need for treatment), and *severe* (loss of tooth structure affecting the enamel and dentine, replacement of hard tissues with atypical restorations, and tooth extraction due to hypomineralization)	28.4
Silva et al. (2020) (Brazil)	Lygidakis et al.^134^	*Mild* (demarcated enamel opacities without enamel breakdown, occasional sensitivity to external stimuli but not brushing and only mild aesthetic concerns on discoloration of the incisors), and **severe** (demarcated enamel opacities with breakdowns, caries, persistent/spontaneous hypersensitivity affecting function and finally strong aesthetic concerns that may have socio-psychological impact)	22.6
Silva Júnior et al. (2015) (Brazil)	Mathu-Muju and WrightWetzel and Reckel scale^130^	*Mild* (Opacities delimited in areas free of occlusal forces, isolated opacities, no enamel loss in opaque areas, no history of dental hypersensitivity, no activities related to caries of affected enamel, alterations of incisors), *moderate* (atypical and intact restorations may be present, opacities delimited in the occlusal/incisal third of the tooth, without loss of the structure after eruption, loss of post-eruptive enamel and carious lesions that are limited to 1 or 2 areas, without participation of cusps, tooth sensitivity and often, aesthetic complaints) and *severe* (post-eruptive losses, history of tooth sensitivity, extensive carious lesions associated with the affected enamel, coronary destruction with pulp involvement, presence of defects in atypical restorations, aesthetic complaints)	21.5
Thakur et al. (2020) (India)	Wetzel and Reckel scale^34^	*Degree 1* (isolated hypomineralization of white cream to yellow–brown color, solely located in the uppermost part of the tooth crown (chewing surface), no post-eruptive enamel breakdown); *degree 2* (enamel hypomineralization of yellow–brown color affecting almost all humps in the coronal part of the tooth crown combined with a small amount of post-eruptive enamel breakdown), and *degree 3* (extensive enamel hypomineralization of yellow–brown color along with extensive post-eruptive enamel breakdown causing changes of the tooth crown morphology)	29.2
Yi et al. (2020) (China)	Jalevik et al.^139^	*Mild* (demarcated enamel opacities without enamel breakdown), and *severe* (demarcated enamel opacities with post-eruptive enamel breakdown, atypical caries, atypical restoration, and missing due to MIH)	39.1
Zawaideh et al. (2011) (Jordania)	Wetzel and Reckel scale^34^	*Degree 1* (isolated hypomineralization of white cream to yellow–brown color, solely located in the uppermost part of the tooth crown (chewing surface), no post-eruptive enamel breakdown); *degree 2* (enamel hypomineralization of yellow–brown color affecting almost all humps in the coronal part of the tooth crown combined with a small amount of post-eruptive enamel breakdown), and *degree 3* (extensive enamel hypomineralization of yellow–brown color along with extensive post-eruptive enamel breakdown causing changes of the tooth crown morphology)	56.0
Villanueva-Gutierrez et al. (2019) (Mexico)	–	*Mild* (demarcated opacities affected less than one-third of the tooth surface, without post-eruptive enamel breakdown), *moderate* (demarcated opacities that affected at least one-third but less than two-thirds of the surface, without post-eruptive enamel breakdown; atypical caries lesions could affect less than two-thirds of the surface), and *severe* (demarcated opacities that affected more than two-thirds of the tooth surface, or the presence of post-eruptive enamel breakdown, atypical caries lesions larger than two-thirds of the surface, or large restaurations with unusual shape, extended to smooth surfaces, or extraction of the tooth because of MIH)	81.5
Negre-Barber et al. (2016) (Spain)	–	*Mild* (white, creamy/yellow or dark brown opacities were counted as mild MIH/HSPM), and *severe* (post-eruptive enamel breakdown, extensive caries with surrounding opacities and atypical restorations, crowns or extractions due to MIH were counted as severe MIH/HSPM)	28.0
Fernandes et al. (2021) (Brazil)	Ghanim et al.^3^	*Mild* (only color changes—cream, white, yellow, orange, or brown), and *severe* (fracture and/or atypical restoration/atypical caries/loss due to MIH)	41.7

*NR* Not reported, *NI* No information, *EAPD* European Academy of Pediatric Dentistry^1^, *mDDE* modified Developmental Defects of Enamel index.

### Sex and geographic location (secondary outcomes)

We further analyzed whether the prevalence results were influenced by study sample size, female/male ratio, geographic location (latitude and longitude) and year of publication (Table 4).

**Table 4:** Meta-regression analyses on the effect of female/male ratio (FMR), latitude, longitude and year. Values are provided as estimate (Standard Error) [Variance explained (%)].

Condition	Sample Size	p-value	FMR	p-value	Latitude	p-value	Longitude	p-value	Year	p-value
MIH	− 0.00 (0.00) [12.5]	< 0.001*	− 0.46 (0.37) [0.0]	0.225	− 0.00 (0.00) [0.0]	0.794	− 0.00 (0.00) [0.0]	0.211	− 0.03 (0.01) [0.0]	0.066
Number of affected molars (%)										
1	0.00 (0.00) [0.0]	0.284	− 0.93 (0.99) [0.0]	0.344	− 0.02 (0.01) [0.0]	0.068	− 0.00 (0.00) [0.0]	0.332	− 0.09 (0.04) [11.6]	0.023*
2	− 0.00 (0.00) [0.0]	0.863	− 0.13 (0.48) [0.0]	0.790	− 0.00 (0.01) [0.0]	0.890	− 0.00 (0.00) [0.0]	0.920	0.02 (0.02) [0.0]	0.301
3	− 0.00 (0.00) [0.0]	0.963	0.56 (0.57) [0.0]	0.327	0.00 (0.01) [0.0]	0.897	− 0.00 (0.00) [0.0]	0.629	0.03 (0.02) [0.0]	0.209
4	− 0.00 (0.00) [0.0]	0.227	1.31 (1.28) [0.0]	0.308	0.01 (0.01) [0.0]	0.302	0.00 (0.00) [0.0]	0.139	0.06 (0.04) [0.0]	0.120
Cases with affected incisors	− 0.00 (0.00) [0.0]	0.433	− 1.03 (1.05) [0.0]	0.325	0.01 (0.01) [0.0]	0.584	0.00 (0.00) [0.0]	0.633	0.02 (0.06) [0.0]	0.694
Cases with both molars and incisors affected	− 0.00 (0.00) [0.0]	0.478	− 0.85 (0.96) [0.0]	0.376	− 0.00 (0.00) [0.0]	0.074	0.00 (0.00) [0.0]	0.915	0.10 (0.03) [0.0]	0.052
HSPM	− 0.00 (0.00) [0.0]	0.116	0.10(2.51) [0.0]	0.966	− 0.00 (0.02) [0.0]	0.932	− 0.01 (0.01) [0.0]	0.338	− 0.16 (0.18) [0.0]	0.394

*MIH* Molar-Incisor Hypomineralization, *HSPM* Hypomineralization of the Second Primary Molars, *95%CI* 95% Confidence Interval, *FMR* Female/Male Ratio. *Significant p-value < 0.05.

Overall, MIH was influenced by the study sample size explaining 7.7% of the accounted heterogeneity, respectively. The year of publication (estimate = − 0.09, p = 0.023) demonstrated a slight influence on the prevalence of MIH cases with one molar affected (explained 11.6% of heterogeneity).

**Figure 2:**
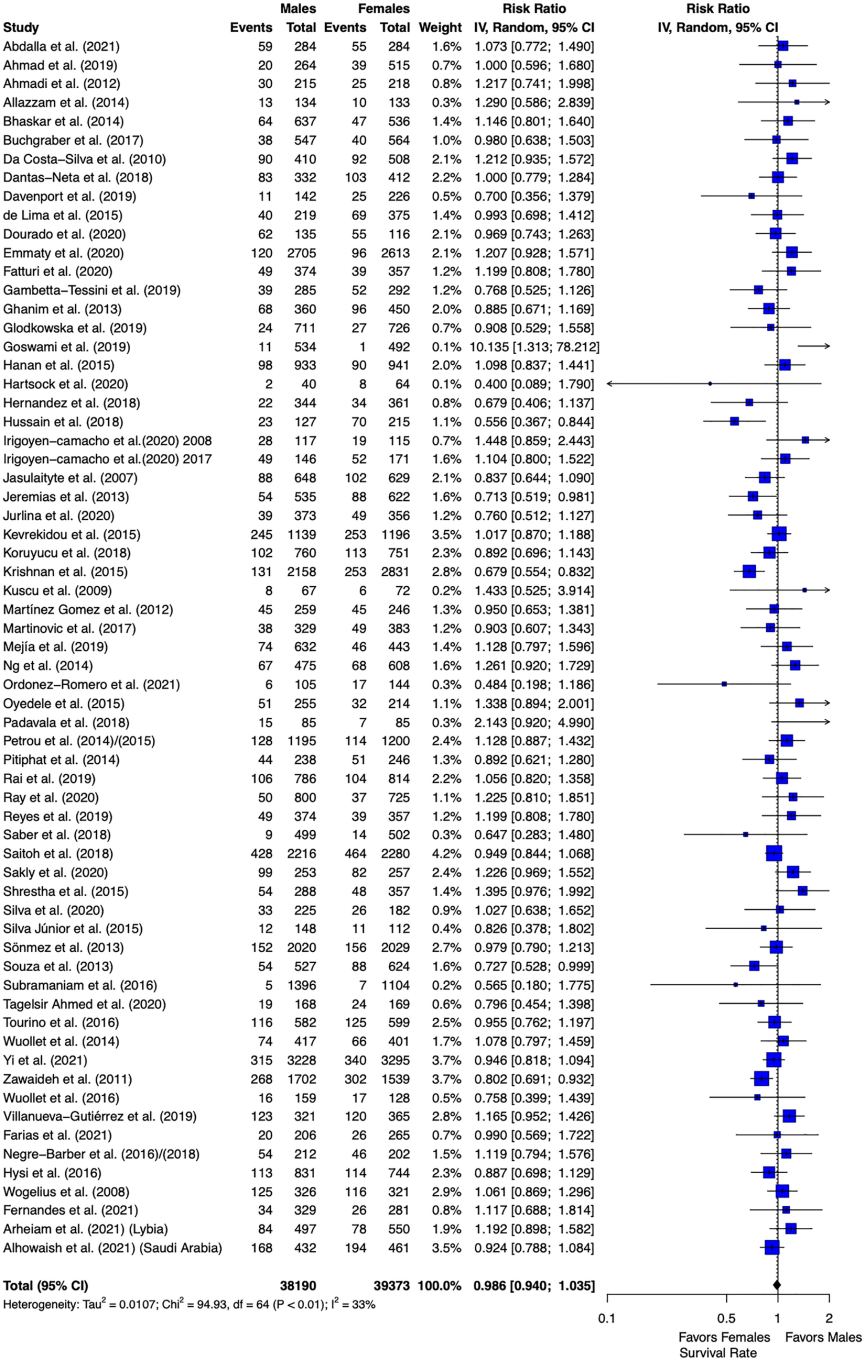
Forest plot of meta-analysis comparing MIH prevalence of female versus male participants.

We then explored whether the prevalence between males and females would differ regarding MIH. Meta-analysis confirmed the latter result from meta-regression that MIH is not sex-related and females and males present a non-significant difference on the prevalence of MIH (0.986, 95% CI 0.940–1.035, I^2^ = 32.6%, p = 0.564) (Fig. 2).

We further explored the prevalence of MIH per continent (Table 5). Among the five continents analyzed, America was the continent with highest prevalence (15.3, 95% CI 12.8–18.3, p < 0.001, I^2^ = 96.3%) and Asia had the lowest prevalence (10.7, 95% CI 78.5–13.5, p < 0.001, I^2^ = 98.7%). The differences between continents (test for subgroup differences) were not significant (p = 0.1643).

**Table 5:** Meta-analysis on the prevalence of MIH per continent.

Continent	N	Estimate	95% CI	p-value	I^2^ (%)
Africa	5	14.5	7.7–25.6	< 0.001	98.1
Asia	29	10.7	8.5–13.5	< 0.001	98.7
America	30	15.3	12.8–18.3	< 0.001	96.3
Europe	34	14.4	12.1–17.1	< 0.001	97.8
Oceania	1	14.7	11.2–18.9	–	–
Test for subgroup differences (random effects model)				p-value = 0.1643	

*MIH* Molar-Incisor Hypomineralization, *HSPM* Hypomineralization of the Second Primary Molars, *95%CI* 95% Confidence Interval, *FMR* female/male ratio.

### Additional analyses

No publication bias was detected in the overall analysis (Table 2), except for the prevalence of cases with one molar affected (p = 0.004).

Using the SORT recommendation, we concluded the estimates obtained are classified as SORT A, that means, the results provide high level of confidence.

## Discussion

### Summary of main findings

The results of the present systematic review estimated a pooled prevalence of MIH at 13.5%. The moderate to severe cases of MIH were estimated at 36.3% of all cases. Having three molars affected with MIH is the least probable situation and affected incisors were seen in 36.6% of the cases. The prevalence of HSPM in MIH cases was estimated at 3.6%. The sample size was a significant source of heterogeneity for the overall MIH prevalence and the year of publication for the prevalence of one molar affected. Sex, year of publication and geographic location were not deemed influential factors in almost all the results. Continents showed no different prevalence on MIH, with the American continent displaying the highest prevalence and the Asian continent the lowest.

### Quality of the evidence and potential biases in the review process

Overall, these results were categorized with a SORT A recommendation, which means that all studies found coherent conclusions regarding the prevalence of MIH and that these results are consistent and good-quality patient-oriented evidence. Furthermore, this is the first systematic review providing pooled estimates on molars and incisors affected with MIH and HSPM cases.

As previously presented, two previous systematic reviews have focused on the prevalence of MIH. Overall, our results provided similar prevalence to the one reported by Schwendicke et al.^5^ (13.1%) and slightly above from Zhao et al.^6^ (14.2%). However, comparing with the latter, the present systematic review expanded the number of countries (49), confirmed the downgrading of alternative case definition of MIH to the overall pooled estimate (while the previous reviews combined classifications), and present new prevalence estimates concerning clinical characteristics of MIH (molas and incisors affected, severity and HSPM).

Regarding the comparison between sexes, our result fully align with those by Schwendicke et al.^5^ (OR 0.92; 0.81–1.04) and Zhao et al.^6^ (regression estimate = 0.005, p-value = 0.938), which means that both girls and boys present similar distribution of MIH lesions.

When analyzing the prevalence among continents, the comparison with literature is not reasonable as we only accounted for the EAPD classification, and this explains why Oceania had no studies available (despite two publication by Mahoney et al.^118,119^). Also, in Zhao et al.^6^, Africa was the continent with lowest prevalence, yet in our review Asia had the lowest prevalence. The American continent includes for the first time studies from the United States of America and Mexico which may explain a decrease in MIH prevalence from the two previous studies, however remains as the continent (super-region) with highest prevalence.

Regarding the methodological aspects, by comparing the EAPD with alternative diagnostic methods as a subgroup analysis we confirmed the downgrading potential of alternative methods to the overall estimates. Thus, this step methodological assortment into the analyses despite the substantial heterogeneity from the meta-analytical estimations. Also, our analyses on the severity, teeth affected and HSPM were severely reduced because this sort of data is still scarce. Future studies shall provide extensive information on these characteristics to confirm these results. Also, we were unable to explore hypothetical MIH-related factors (both medical, sociodemographic and environmental) once again because of the lack of relevant information, and this should be taken into account in future epidemiological studies.

All in all, readers must bear in mind that although the overall prevalence seems to be constant over the time, new prevalence data has been pooled that contribute to understand the clinical characteristics of this enamel defect entity.

### Strengths and potential limitations

This systematic review was conducted following PRISMA a strict guideline for data reporting, a comprehensive literature search and a meticulous predefined protocol. Furthermore, prior to any analysis, we compared the EAPD case definition with other classifications than the EAPD, and we confirmed substantial differences with a downgrading in prevalence when alternative methods were applied. We have attempted to explore ways to mitigate heterogeneity, and all studies used to compute estimates (and that employed the EAPD case definition) were of high methodological quality. Another advantage of this study is that we have expanded the search for potential sources of heterogeneity with the addition of geographic measures and the further assessment into the new prevalence estimates. Also, the number of included participants has increase, which is logical given the increase in studies included, yet this is a point to keep in mind.

Nevertheless, there are a number of limitations important discussing. Almost half of the studies had not fulfilled the criterion of representativeness and this is a point where future studies shall be careful. These results should be prudently analyzed because of the elevated heterogeneity observed in some of the reported estimates, though from our analyses the heterogeneity mostly derives from the variability between regions already discussed in a previous study assessing meta-analysis of prevalence^131^, rather than the sources of heterogeneity considered as proven through meta-regression.

Also, a number of studies have not employed the EAPD case definition for MIH and after the subgroup analysis aforementioned they were not accounted for the analyses. It is essential that there is a standardization of the classification used, which is a topic already widely discussed in the literature^132^. Ergo, and given the results of the present systematic review, several challenges may emerge. First, a global partnership between all geographic representative associations shall be attained, to ultimately ensure a standardization of MIH reporting and, certainly, will encourage new and updated epidemiological and clinical data. Second, this suggested consensus will clarify the terminologies and guidelines towards a global alliance that will benefit all people affected by MIH. All in all, these may contribute to overcoming the lack of epidemiological data and a still methodologically unsettled reporting approach.

Only a percentage of the overall included studies reported data on the teeth affected with MIH, the severity of cases or HSPM cases. Several classifications for the severity of MIH have been proposed^133,134^, and some date before the EAPD 2003, such as Leppäniemi^135^ or the Wetzel & Reckel scale^117,136^. Moreover, the MIH Treatment Need Index (MIH-TNI) was recently presented, which is^137^ part of the Wuerzburg MIH concept. Nevertheless, the lack of a homogeneous definition may have contributed to the heterogeneity of results, making it urgent to establish a consensual severity classification.

Hence, future studies should focus on data on these prevalence characteristics to deepen our knowledge regarding the specifics of MIH. These information are of the utmost relevance for clinicians and may aid the development and implementation of future oral health programs.

## Conclusion

The estimated prevalence of MIH was estimated at 13.5%. Moderate to severe cases of MIH were estimated at 36.3%. Affected incisors were seen in 36.6% of the cases. The prevalence of hypomineralization of the second primary molars in MIH cases was estimated at 3.6%. Overall, these results were categorized with a SORT A recommendation.

## Supplementary Information

Supplementary Information.

## Data Availability

Data is provided in the materials of the paper.
