# Supplementary material for: The prevalence of molar-incisor hypomineralization: a systematic review and meta-analysis
Source: Sci Rep. 2021 Nov 17;11:22405. doi: 10.1038/s41598-021-01541-7 (PMC8599453; doi:10.1038/s41598-021-01541-7)

**Online Supplemental Information**

**The Prevalence of Molar-Incisor Hypomineralization: A Systematic Review and Meta-Analysis**

Luísa Bandeira Lopes ^1^, Vanessa Machado ^1,2^, Paulo Mascarenhas ^1,2^, José João Mendes ^1,2^, João Botelho ^1,2^

^1^ Clinical Research Unit (CRU), Centro de Investigação Interdisciplinar Egas Moniz, Egas Moniz – Cooperativa de Ensino Superior, CRL, 2829-511 Almada, Portugal

^2^ Evidence-Based Hub, Centro de Investigação Interdisciplinar Egas Moniz, Egas Moniz – Cooperativa de Ensino Superior, CRL, 2829-511 Almada, Portugal

**Running title**: Global prevalence of MIH

**Corresponding Author:**

João Botelho

Clinical Research Unit (CRU), Egas Moniz Interdisciplinary Research Center (CiiEM)

Egas Moniz – Cooperativa de Ensino Superior

Address: Campus Universitário, Quinta da Granja, Monte de Caparica, 2829 - 511 Caparica, Almada, Portugal

Phone: (+351) 212 946 737; Fax: not available; E-mail: [jbotelho@egasmoniz.edu.pt](mailto:jbotelho@egasmoniz.edu.pt)

**CONFLICT OF INTEREST**

The authors declare no conflict of interest.

**ACKNOWLEDGEMENTS**

Nothing to declare

Supplementary S1. PRISMA 2009 Checklist

| **Section/topic** | **#** | **Checklist item** | **Reported on page #** |
| --- | --- | --- | --- |
| **TITLE** | | |  |
| Title | 1 | Identify the report as a systematic review, meta-analysis, or both. | 1 |
| **ABSTRACT** | | |  |
| Structured summary | 2 | Provide a structured summary including, as applicable: background; objectives; data sources; study eligibility criteria, participants, and interventions; study appraisal and synthesis methods; results; limitations; conclusions and implications of key findings; systematic review registration number. | 2 |
| **INTRODUCTION** | | |  |
| Rationale | 3 | Describe the rationale for the review in the context of what is already known. | 3 |
| Objectives | 4 | Provide an explicit statement of questions being addressed with reference to participants, interventions, comparisons, outcomes, and study design (PICOS). | 3 |
| **METHODS** | | |  |
| Protocol and registration | 5 | Indicate if a review protocol exists, if and where it can be accessed (e.g., Web address), and, if available, provide registration information including registration number. | 3-4 |
| Eligibility criteria | 6 | Specify study characteristics (e.g., PICOS, length of follow-up) and report characteristics (e.g., years considered, language, publication status) used as criteria for eligibility, giving rationale. | 4 |
| Information sources | 7 | Describe all information sources (e.g., databases with dates of coverage, contact with study authors to identify additional studies) in the search and date last searched. | 4 |
| Search | 8 | Present full electronic search strategy for at least one database, including any limits used, such that it could be repeated. | 4 |
| Study selection | 9 | State the process for selecting studies (i.e., screening, eligibility, included in systematic review, and, if applicable, included in the meta-analysis). | 4-5 |
| Data collection process | 10 | Describe method of data extraction from reports (e.g., piloted forms, independently, in duplicate) and any processes for obtaining and confirming data from investigators. | 5 |
| Data items | 11 | List and define all variables for which data were sought (e.g., PICOS, funding sources) and any assumptions and simplifications made. | 5 |
| Risk of bias in individual studies | 12 | Describe methods used for assessing risk of bias of individual studies (including specification of whether this was done at the study or outcome level), and how this information is to be used in any data synthesis. | 5 |
| Summary measures | 13 | State the principal summary measures (e.g., risk ratio, difference in means). | 5-6 |
| Synthesis of results | 14 | Describe the methods of handling data and combining results of studies, if done, including measures of consistency (e.g., I^2^) for each meta-analysis. | 5-6 |

| **Section/topic** | **#** | **Checklist item** | **Reported on page #** |
| --- | --- | --- | --- |
| Risk of bias across studies | 15 | Specify any assessment of risk of bias that may affect the cumulative evidence (e.g., publication bias, selective reporting within studies). | NA |
| Additional analyses | 16 | Describe methods of additional analyses (e.g., sensitivity or subgroup analyses, meta-regression), if done, indicating which were pre-specified. | 6 |
| **RESULTS** | | |  |
| Study selection | 17 | Give numbers of studies screened, assessed for eligibility, and included in the review, with reasons for exclusions at each stage, ideally with a flow diagram. | 6 |
| Study characteristics | 18 | For each study, present characteristics for which data were extracted (e.g., study size, PICOS, follow-up period) and provide the citations. | 6 |
| Risk of bias within studies | 19 | Present data on risk of bias of each study and, if available, any outcome level assessment (see item 12). | 6 |
| Results of individual studies | 20 | For all outcomes considered (benefits or harms), present, for each study: (a) simple summary data for each intervention group (b) effect estimates and confidence intervals, ideally with a forest plot. | 6 |
| Synthesis of results | 21 | Present results of each meta-analysis done, including confidence intervals and measures of consistency. | 6-7 |
| Risk of bias across studies | 22 | Present results of any assessment of risk of bias across studies (see Item 15). | NA |
| Additional analysis | 23 | Give results of additional analyses, if done (e.g., sensitivity or subgroup analyses, meta-regression [see Item 16]). | 7 |
| **DISCUSSION** | | |  |
| Summary of evidence | 24 | Summarize the main findings including the strength of evidence for each main outcome; consider their relevance to key groups (e.g., healthcare providers, users, and policy makers). | 7 |
| Limitations | 25 | Discuss limitations at study and outcome level (e.g., risk of bias), and at review-level (e.g., incomplete retrieval of identified research, reporting bias). | 7-9 |
| Conclusions | 26 | Provide a general interpretation of the results in the context of other evidence, and implications for future research. | 9 |
| **FUNDING** | | |  |
| Funding | 27 | Describe sources of funding for the systematic review and other support (e.g., supply of data); role of funders for the systematic review. | 9 |

NA – Not applicable

*From:*  Moher D, Liberati A, Tetzlaff J, Altman DG, The PRISMA Group (2009). Preferred Reporting Items for Systematic Reviews and Meta-Analyses: The PRISMA Statement. PLoS Med 6(7): e1000097. doi:10.1371/journal.pmed1000097

For more information, visit: **www.prisma-statement.org**.

Supplementary S2. List of potentially relevant studies not included in the systematic review, along with the reasons for exclusion.

| No. | Reference | Reason |
| --- | --- | --- |
| 1 | Anood Alshehhi, Manal Al Halabi, Iyad Hussein, Anas Salami, Amar Hassan& Mawlood Kowash. Enamel defects and caries prevalence in preterm children aged 5-10 years in Dubai. Libyan Journal of Medicine, 2020, Vol. 15 | Enamel defects without MIH data |
| 2 | Bahrololoomi Z, Amrollahi N, Mostafaloo N. The prevalence and extent of Molar-Incisor-Hypo-mineralization by gender in a group of Iranian Children. Iran J Public Health, Aug 2020, Vol. 49, No.8, pp.1585-1587 | Letter to the Editor |
| 3 | Głódkowska N, Emerich K. The impact of environmental air pollution on the prevalence of molar incisor hypo­ mineralization in schoolchildren: A cross­sectional study. Adv Clin Exp Med. 2020;29(12):1469–1477. | Previously studied sample |
| 4 | Kevrekidou A, Kosma I, Kotsanos I, Arapostathis KN, Kotsanos N. Enamel opacities in all other than Molar Hypomineralization index teeth of adolescents. Int J Paediatr Dent. 2020, Oct 1 | No prevalence data |
| 5 | Tseveenjav B, Furuholm J, Mulic A, Valen H, Maisala T, Turunen S, Varsio S, Auero M, Tjäderhane L. Estimating molar-incisor-hypomineralization among 8-year-olds based on 15-year public oral health practice-based data. 2020 Oct;78(7):535-540 | No prevalence data |
| 6 | Wogelius P, Viuff JH, Haubek D. Use of asthma drugs and prevalence of molar incisor hypomineralization. Int J Paediatr Dent. 2020;00:1–7. | Study on asthma patients |
| 7 | Vieira AR. Prevalence of molar incisor hypomineralisation has a North–South gradient between Europe and North Africa. Eur Arch Paediatr Dent. 2019 Oct;20(5):501-502 | Letter to the Editor |
| 8 | Ardini YD, Ismail NN, Azni NDM, Harun NA. Molar incisor hypomineralization prevalence and associated risk factors among children at the polyclinic, Kulliyyah of dentistr, IIUM. Materials today, 2019;16:2351-2356 | Study of perinatal and post-natal samples |
| 9 | Condò R, Perugia C, Maturo P, Docimo R. MIH: epidemiologic clinic study in paediatric patient. Oral Implantol (Rome). 2012 Apr;5(2-3):58-69. Epub 2012 Nov 16. PMID: 23285408; PMCID: PMC3505102. | Unreferred MIH diagnostic criteria |
| 10 | Ravindran R, Saji AM. Prevalence of the development defects of the enamel in children aged 12-15 years in Kollan district. J Int Soc Prev Community Dent. Jan-Feb 2016;6(1):28-33 | Enamel defects without MIH data |
| 11 | Salem K, Aziz D, Asadi M. Prevalence and Predictors of Molar Incisor Hypomineralization (MIH) among Rural Children in Northern Iran. Iran J Public Health 2016 Nov;45(11):1528-1530. | Letter to the Editor |
| 12 | Kosma I, Kevrekidou A, Boka V, Arapostathis K, Kotsanos N. Molar incisor hypomineralisation (MIH): correlation with dental caries and dental fear. Eur Arch Paediatric Dent. 2016 Apr;17(2):123-9 | No prevalence data |
| 13 | Lang J, Birkenbeil S, Bock S, Heinrich-weltzien R, Kromeyer-Hauschild K. Dental enamel defects in German mediaval and early-modern-age populations. J. Biol.Clin. Anthropol. 2016, :73/4;343-354 | Prevalence of MIH in human skeletal remains |
| 14 | Tunc ES, Ulusoy AT, Bayrak S, Cankaya S.. Dental development in children with severe molar-incisor hypomineralization in Samsun, Turkey. 2013, Journal of Oral Science, Vol. 55, No. 3, 203-207 | No prevalence data |
| 15 | Ghanim A, Manton D, Bailey D, Mariño R, Morgan M. Risk factors in the occurrence of molar-incisor hypomineralization amongst a group of iraqui children. Int J Paediatr Dent. 2013 May; 23(3):197-206 | Risk factors in the occurrence of MIH |
| 16 | Li L, Li J. [Investigation of molar-incisor hypomineralization among children from 6 to 11 years in Lucheng district, Wenzhou city]. Shanghai Kou Qiang Yi Xue. 2012 Oct;21(5):576-9. Chinese. PMID: 23135192. | Unable to access |
| 17 | Kukleva MP, Petrova SG, Kondeva VK, Nihtyanova TI. Folia Med (Plovdiv). 2008 Jul-Sep;50(3):71-5. | Unable to access |
| 18 | Muratbegovic A, Zukanovic A, Markovic N. Molar-Incisor-Hypomineralisation impact on developmental defects of enamel prevalence in a low fluoridated area. European Archives of Paediatric dentistry 2008;9(4) | Enamel defects without MIH data |
| 19 | Preusser SE, Ferring V, Wleklinski C, Wetzel W-E. c Health Dent Summer 2007;67(3):148-50 | Short Communication |
| 20 | Balmer RC, Laskey D, Mahoney E, Toumba KJ. Prevalence of enamel defects and MIH in non-fluoridated and fluoridated communities. Eur J Paediatr Dent. 2005 Dec;6(4):209-12. PMID: 16426121. | No prevalence data |
| 21 | Arrow P. Prevalence of developmental enamel defects of the first permanent molars among school children in Western Australia. Aust Dent J. 2008 Sep;53(3):250-9. doi: 10.1111/j.1834-7819.2008.00057.x. PMID: 18782370. | Enamel defects without MIH data |
| 22 | Opydo-Szymaczek J, Gerreth K. Developmental Enamel Defects of the Permanent First Molars and Incisors and Their Association with Dental Caries in the Region of Wielkopolska, Western Poland. Oral Health Prev Dent. 2015;13(5):461-9. doi: 10.3290/j.ohpd.a33088. PMID: 25431802. | Enamel defects without MIH data |

Supplementary S3. Newcastle-Ottawa Scale assessment.

| Study | SELECTION | | | | COMPARABILITY | EXPOSURE | | | RoB Score |
| --- | --- | --- | --- | --- | --- | --- | --- | --- | --- |
|  | Is the case definition adequate? | Representativeness of the cases? | Selection of controls? | Definition of controls? | Comparability of cases and controls of design or analysis? | Ascertainment of exposure? | Same method of ascertainment for cases and controls | Non-response rate? |  |
| Abdalla et al. (2021) | a | b | a | a | a/b | a | a | a | 8 (Low) |
| Ahmad et al. (2019) | a | b | a | a | a/b | a | a | a | 8 (Low) |
| Ahmadi et al. (2012) | a | b | a | a | a/b | a | a | a | 8 (Low) |
| Alhowaish et al. (2021) | a | a | a | a | a/b | a | a | a | 9 (Low) |
| Allazzam et al. (2014) | a | a | a | a | a/b | a | a | a | 9 (Low) |
| Amend et al. (2020) | a | a | a | a | a/b | a | a | a | 9 (Low) |
| Arheiam et al. (2021) | a | b | a | a | a/b | a | a | a | 8 (Low) |
| Arslanagic-Muratbegovic et al. (2020) | a | b | a | a | a/b | a | a | a | 8 (Low) |
| Balmer et al. (2011)/(2015) | a | a | a | a | a/b | a | a | a | 9 (Low) |
| Bhaskar et al. (2014) | a | a | a | a | a/b | a | a | a | 9 (Low) |
| Biondi et al. (2011) | a | a | a | a | a/b | a | a | a | 9 (Low) |
| Biondi et al. (2012) | a | a | a | a | a/b | a | a | a | 9 (Low) |
| Buchgraber et al. (2017) | a | b | a | a | a/b | a | a | a | 8 (Low) |
| Calderara et al. (2005) | a | b | a | a | a/b | a | a | a | 8 (Low) |
| Cho et al. (2008) | a | b | a | a | a/b | a | a | a | 8 (Low) |
| Da Costa-Silva et al. (2010) | a | a | a | a | a/b | a | a | a | 9 (Low) |
| Dantas-Neta et al. (2016) | a | a | a | a | a/b | a | a | a | 9 (Low) |
| Dantas-Neta et al. (2018) | a | b | a | a | a/b | a | a | a | 8 (Low) |
| Davenport et al. (2019) | a | a | a | a | a/b | a | a | a | 9 (Low) |
| de Lima et al. (2015) | a | a | a | a | a/b | a | a | a | 9 (Low) |
| Dietrich et al. (2003) | a | b | a | a | a | a | a | a | 7 (Low) |
| Dourado et al. (2020) | a | a | a | a | a/b | a | a | a | 9 (Low) |
| Elfrink et al. (2012) | a | a | a | a | a/b | a | a | a | 9 (Low) |
| Elzein et al. (2019) | a | a | a | a | a/b | a | a | a | 9 (Low) |
| Emmaty et al. (2020) | a | b | a | b | a/b | a | a | a | 7 (Low) |
| Farias et al. (2021) | a | a | a | a | a/b | a | a | a | 9 (Low) |
| Fatturi et al. (2020) | a | a | a | a | a/b | a | a | a | 9 (Low) |
| Fernandes et al. (2021) | a | a | a | a | a/b | a | a | a | 9 (Low) |
| Freitas Fernandes et al. (2021) | a | a | a | a | a/b | a | a | a | 9 (Low) |
| Fteita et al. (2006) | a | b | a | a | a/b | a | a | a | 8 (Low) |
| Gambetta-Tessini et al. (2018) | a | a | a | a | a/b | a | a | a | 9 (Low) |
| Gambetta-Tessini et al. (2019) | a | b | a | a | a/b | a | a | a | 8 (Low) |
| Garcia-Margarit et al. (2013) | a | b | a | a | a/b | a | a | a | 8 (Low) |
| Ghanim et al. (2011) | a | a | a | a | a/b | a | a | a | 9 (Low) |
| Ghanim et al. (2013) | a | b | a | a | a/b | a | a | a | 8 (Low) |
| Glodkowska et al. (2019) | a | b | a | a | a/b | a | a | a | 8 (Low) |
| Goswami et al. (2019) | a | b | a | a | a | a | a | a | 7 (Low) |
| Groselj et al. (2013) | a | b | a | a | a/b | a | a | a | 8 (Low) |
| Gurrusquieta et al. (2017) | a | b | a | a | a/b | a | a | a | 8 (Low) |
| Hanan et al. (2015) | a | b | a | a | a/b | a | a | a | 8 (Low) |
| Hartsock et al. (2020) | a | a | a | a | a/b | a | a | a | 9 (Low) |
| Heitmuller et al. (2013) | a | a | a | a | a/b | a | a | a | 9 (Low) |
| Hernandez et al. (2018) | a | b | a | a | a/b | a | a | a | 8 (Low) |
| Hussain et al. (2018) | a | b | a | a | a/b | a | a | a | 8 (Low) |
| Hussein et al. (2015) | a | a | a | a | a/b | a | a | a | 9 (Low) |
| Hysi et al. (2016) | a | a | a | a | a/b | a | a | a | 9 (Low) |
| Irigoyen-Camacho et al. (2020) | a | b | a | a | a/b | a | a | a | 8 (Low) |
| Janković et al. (2014) | a | a | a | a | a/b | a | a | a | 9 (Low) |
| Jasulaityte et al. (2008) | a | a | a | a | a/b | a | a | a | 8 (Low) |
| Jasulaityte et al. (2008) | a | b | a | a | a/b | a | a | a | 8 (Low) |
| Jeremias et al. (2013) | a | b | a | a | a/b | a | a | a | 8 (Low) |
| Jurlina et al. (2020) | a | b | a | a | a/b | a | a | a | 8 (Low) |
| Kemoli et al. (2009) | a | b | a | a | a/b | a | a | a | 8 (Low) |
| Kevrekidou et al. (2015) | a | b | a | a | a/b | a | a | a | 8 (Low) |
| Kirthiga et al. (2015) | a | a | a | a | a/b | a | a | a | 9 (Low) |
| Kılınç et al. (2019) | a | a | a | a | a/b | a | a | a | 9 (Low) |
| Kohlboeck et al. (2013) | a | b | a | a | a/b | a | a | a | 8 (Low) |
| Koruyucu et al. (2018) | a | b | a | a | a/b | a | a | a | 8 (Low) |
| Krishnan et al. (2015) | a | b | a | a | a/b | a | a | a | 8 (Low) |
| Kühnisch et al. (2018) | a | a | a | a | a | a | a | a | 8 (Low) |
| Kuscu et al. (2009) | a | a | a | a | a/b | a | a | a | 9 (Low) |
| López Jordi et al. (2014) | a | b | a | a | a | a | a | a | 7 (Low) |
| Lygidakis et al. (2008) | a | a | a | a | a/b | a | a | a | 9 (Low) |
| Mahoney et al. (2009) | a | a | a | a | a/b | a | a | a | 9 (Low) |
| Mahoney et al. (2011) | a | a | a | a | a/b | a | a | a | 9 (Low) |
| Martínez Gomez et al. (2012) | a | a | a | a | a/b | a | a | a | 9 (Low) |
| Martinovic et al. (2017) | a | b | a | a | a/b | a | a | a | 8 (Low) |
| Mejía et al. (2019) | a | a | a | a | a/b | a | a | a | 9 (Low) |
| Mishra et al. (2016) | a | b | a | a | a/b | a | a | a | 8 (Low) |
| Mittal et al. (2013) | a | a | a | a | a/b | a | a | a | 9 (Low) |
| Mulic et al. (2017) | a | b | a | a | a/b | a | a | a | 8 (Low) |
| Muratbegovic et al. (2007) | a | b | a | a | a/b | a | a | a | 8 (Low) |
| Negre-Barber et al. (2016)/(2018) | a | b | a | a | a/b | a | a | a | 8 (Low) |
| Ng et al. (2014) | a | b | a | a | a/b | a | a | a | 8 (Low) |
| Ordonez-Romero et al. (2021) | a | b | a | a | a/b | a | a | a | 8 (Low) |
| Oyedele et al. (2015) | a | b | a | a | a/b | a | a | a | 8 (Low) |
| Padavala et al. (2018) | a | b | a | a | a/b | a | a | a | 8 (Low) |
| Parikh et al. (2012) | a | b | a | a | a/b | a | a | a | 8 (Low) |
| Petrou et al. (2014)/(2015) | a | b | a | a | a/b | a | a | a | 8 (Low) |
| Pitiphat et al. (2014) | a | a | a | a | a | a | a | a | 8 (Low) |
| Portella et al. (2019) | a | a | a | a | a/b | a | a | a | 9 (Low) |
| Preusser et al. (2007) | a | b | a | a | a/b | a | a | a | 8 (Low) |
| Rai et al. (2018) | a | a | a | a | a/b | a | a | a | 9 (Low) |
| Rai et al. (2019) | a | a | a | a | a/b | a | a | a | 9 (Low) |
| Ray et al. (2020) | a | a | a | a | a/b | a | a | a | 9 (Low) |
| Reyes et al. (2019) | a | a | a | a | a/B | a | a | a | 9 (Low) |
| Rodrigues et al. (2015) | a | b | a | b | a | a | a | a | 6 (Moderate) |
| Saber et al. (2018) | a | a | a | a | a | a | a | a | 8 (Low) |
| Saitoh et al. (2018) | a | a | a | a | a/b | a | a | a | 9 (Low) |
| Sakly et al. (2020) | a | a | a | a | a/b | a | a | a | 9 (Low) |
| Schmalfuss et al. (2015) | a | a | a | a | a | a | a | a | 8 (Low) |
| Shrestha et al. (2015) | a | a | a | a | a/b | a | a | a | 9 (Low) |
| Sidhu et al. (2019) | a | a | a | a | a | a | a | a | 8 (Low) |
| Silva et al. (2020) | a | b | a | a | a/b | a | a | a | 8 (Low) |
| Silva Júnior et al. (2015) | a | a | a | a | a/b | a | a | a | 9 (Low) |
| Singh et al. (2020) | a | b | a | a | a/b | a | a | a | 8 (Low) |
| Sönmez et al. (2013) | a | b | a | a | a | a | a | a | 7 (Low) |
| Sosa-Soto et al. (2021) | a | b | a | a | a/b | a | a | a | 8 (Low) |
| Souza et al. (2013) | a | b | a | a | a/b | a | a | a | 8 (Low) |
| Soviero et al. (2009) | a | b | a | a | a/b | a | a | b | 7 (Low) |
| Subramaniam et al. (2016) | a | b | a | a | a/b | a | a | a | 8 (Low) |
| Tagelsir Ahmed et al. (2020) | a | a | a | a | a/b | a | a | a | 9 (Low) |
| Temilola et al. (2015) | a | b | a | a | a/b | a | a | a | 8 (Low) |
| Thakur et al. (2020) | a | b | a | a | a/b | a | a | a | 8 (Low) |
| Tourino et al. (2016) | a | a | a | a | a/b | a | a | a | 9 (Low) |
| Villanueva-Gutiérrez et al. (2019) | a | a | a | a | a/b | a | a | a | 9 (Low) |
| Wogelius et al. (2008) | a | a | a | a | a/b | a | a | a | 9 (Low) |
| Wuollet et al. (2014) | a | b | a | a | a/b | a | a | a | 8 (Low) |
| Wuollet et al. (2016) | a | b | a | a | a/b | a | a | a | 8 (Low) |
| Wuollet et al. (2018) | a | b | a | a | a/b | a | a | a | 8 (Low) |
| Yannam et. (2016) | a | a | a | a | a/b | a | a | a | 9 (Low) |
| Yi et al. (2021) | a | a | a | a | a/b | a | a | a | 9 (Low) |
| Zawaideh et al. (2011) | a | a | a | a | a/b | a | a | a | 9 (Low) |

Supplementary S4. Comparison between EAPD Athens 2003 classification versus other classifications (defined as Others).

Number of studies combined: k = 116

proportion 95%-CI

Random effects model 0.1277 [0.1153; 0.1413]

Quantifying heterogeneity:

tau^2 = 0.3940 [0.3615; 0.6442]; tau = 0.6277 [0.6013; 0.8026];

I^2 = 97.9% [97.7%; 98.1%]; H = 6.92 [6.63; 7.22]

Quantifying residual heterogeneity:

I^2 = 97.9% [97.7%; 98.1%]; H = 6.89 [6.60; 7.19]

Test of heterogeneity:

Q d.f. p-value

5503.79 115 0

Results for subgroups (random effects model):

k proportion 95%-CI

classf = EAPD 99 0.1350 [0.1208; 0.1506]

classf = Other 17 0.0914 [0.0702; 0.1182]

tau^2 tau Q I^2

classf = EAPD 0.4022 0.6342 4784.73 98.0%

classf = Other 0.3435 0.5861 626.99 97.4%

Test for subgroup differences (random effects model):

Q d.f. p-value

Between groups 7.52 1 0.0061

Supplementary S5. Forest plot on the prevalence of MIH using the EAPD 2003 case definition.

Supplementary S6. Cumulative meta-analysis (A) and leave-one-out meta-analysis (B), exploring the existence of influential studies.

A) B)

Supplementary S7. Forest plot on the prevalence of moderate-to-severe MIH.

Supplementary S8. Forest plot on the prevalence of one molar affected with MIH.


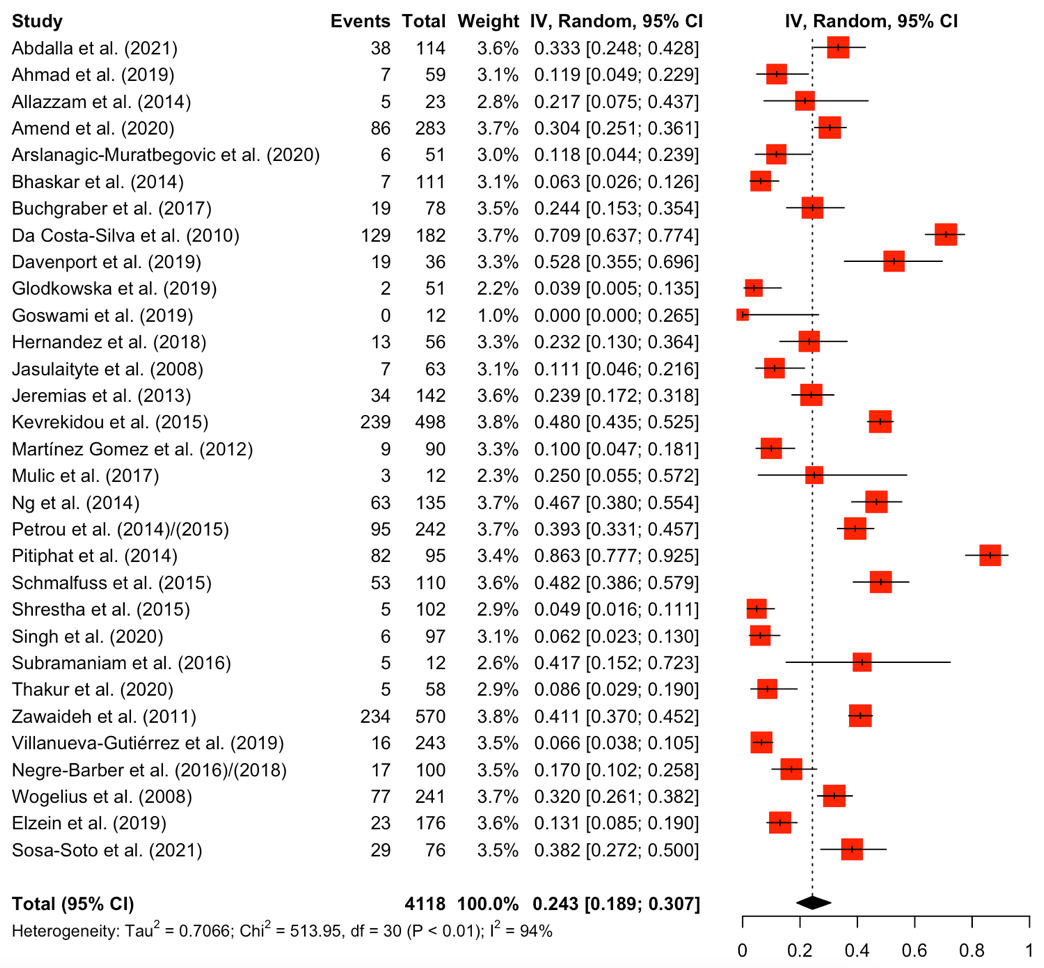


Supplementary S9. Forest plot on the prevalence of two molars affected with MIH.


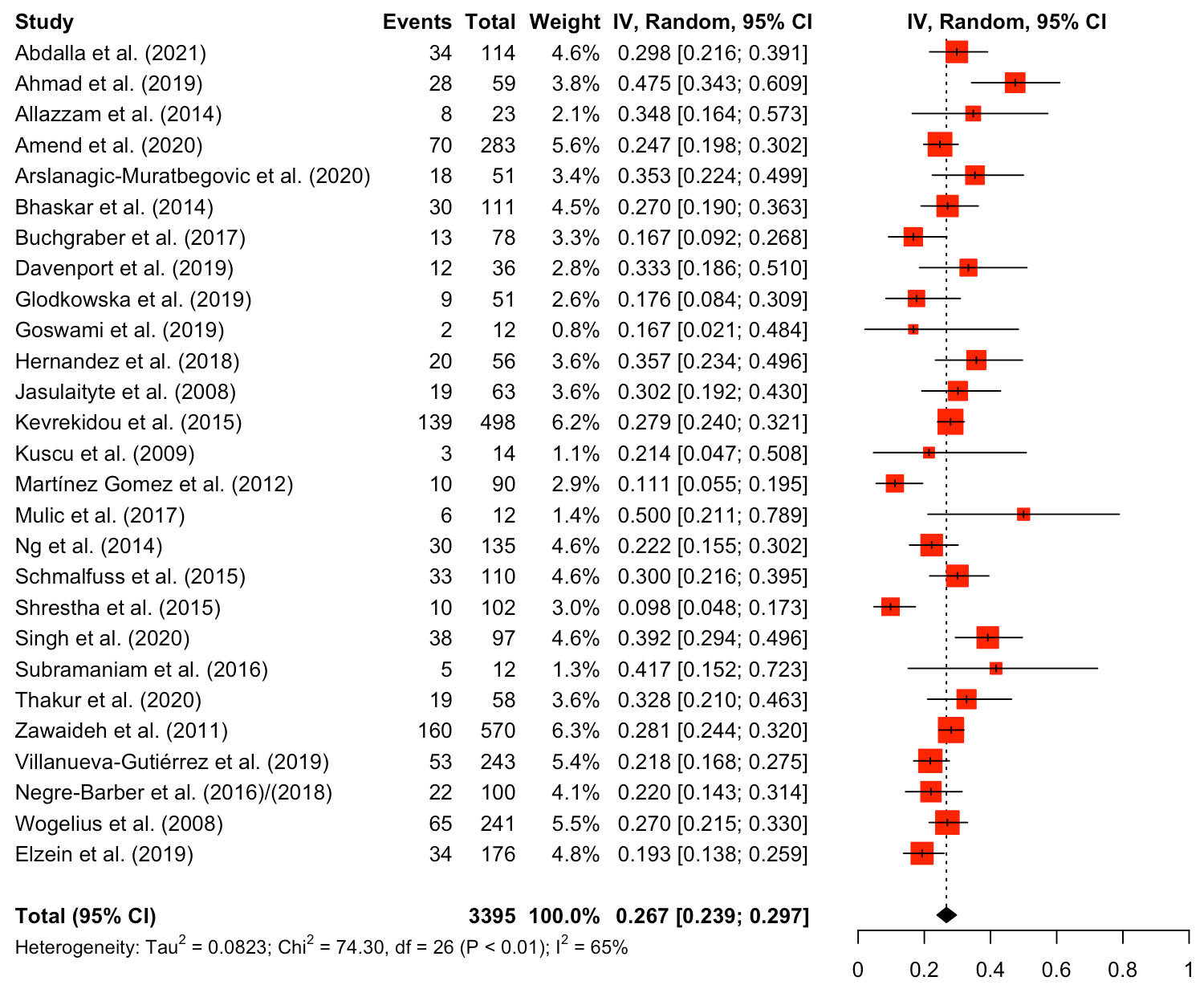


Supplementary S10. Forest plot on the prevalence of three molars affected with MIH.


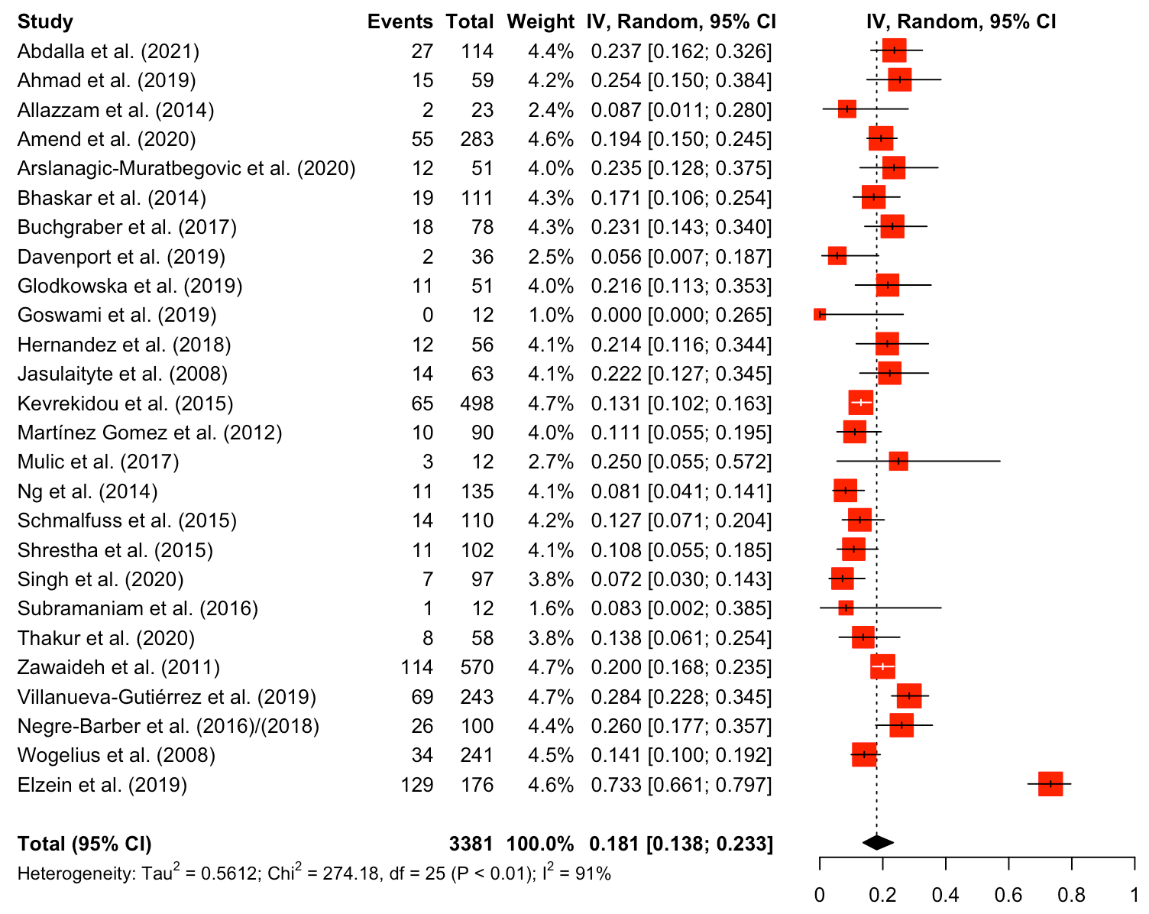


Supplementary S11. Forest plot on the prevalence of four molars affected with MIH.


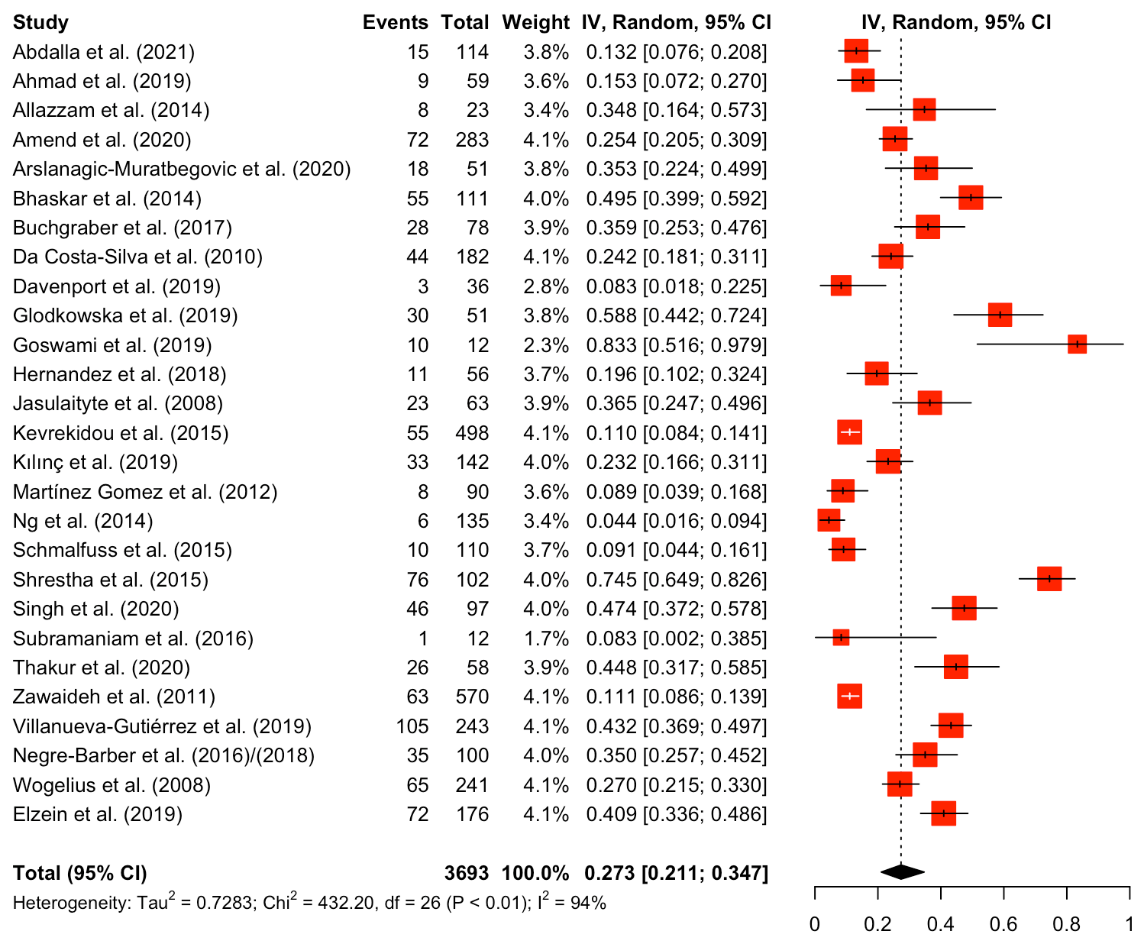


Supplementary S12. Forest plot on the prevalence of incisors affected with MIH.

Supplementary S13. Forest plot on the prevalence of both molars and incisors affected with MIH.

Supplementary S14. Forest plot on the prevalence of hypomineralized second permanent molars.


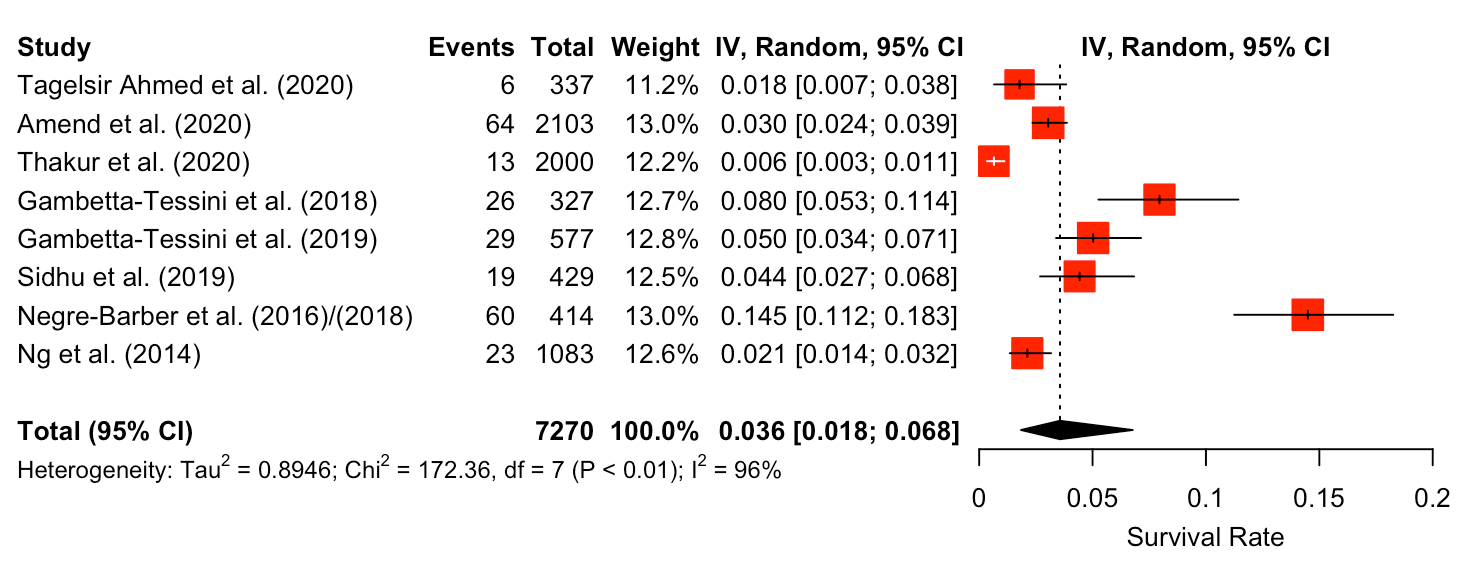

Supplement: Supplementary file 1 — Supplementary Information. [file 41598_2021_1541_MOESM1_ESM.docx]
